# Action simulation: time course and representational mechanisms

**DOI:** 10.3389/fpsyg.2013.00387

**Published:** 2013-07-04

**Authors:** Anne Springer, Jim Parkinson, Wolfgang Prinz

**Affiliations:** ^1^Department of Psychology, Max Planck Institute for Human Cognitive and Brain SciencesLeipzig, Germany; ^2^Department of Sport and Exercise Psychology, University of PotsdamPotsdam, Germany; ^3^Institute of Cognitive Neuroscience, University College LondonLondon, UK; ^4^Sackler Centre for Consciousness Science, University of SussexFalmer, UK

**Keywords:** action simulation, internal forward models, occlusion, point-light action, static matching, predictive coding

## Abstract

The notion of action simulation refers to the ability to re-enact foreign actions (i.e., actions observed in other individuals). Simulating others' actions implies a *mirroring* of their activities, based on one's own sensorimotor competencies. Here, we discuss theoretical and experimental approaches to action simulation and the study of its representational underpinnings. One focus of our discussion is on the timing of internal simulation and its relation to the timing of external action, and a paradigm that requires participants to predict the future course of actions that are temporarily occluded from view. We address transitions between perceptual mechanisms (referring to action representation before and after occlusion) and simulation mechanisms (referring to action representation during occlusion). Findings suggest that action simulation runs in real-time; acting on newly created action representations rather than relying on continuous visual extrapolations. A further focus of our discussion pertains to the functional characteristics of the mechanisms involved in predicting other people's actions. We propose that two processes are engaged, dynamic updating and static matching, which may draw on both semantic and motor information. In a concluding section, we discuss these findings in the context of broader theoretical issues related to action and event representation, arguing that a detailed functional analysis of action simulation in cognitive, neural, and computational terms may help to further advance our understanding of action cognition and motor control.

## Representation of occluded action

In recent years, the concept of *simulation* has flourished within various fields of psychological research, ranging from agency (Ruby and Decety, 2001), action perception (Rizzolatti et al., 1996; Blakemore and Decety, 2001; Mukamel et al., 2010), imitation (Brass et al., 2001; Buccino et al., 2004), mind reading (Gordon, 1996, 2001; Goldman, 2005, 2006), and empathy (Chartrand and Bargh, 1999; Gallese et al., 2004) to the study of clinical issues like schizophrenia (Enticott et al., 2008). Across these domains and the majority of studies, the term simulation concordantly expresses the idea that humans possess non-conceptual and direct ways of understanding others' thoughts, feelings, intentions and actions by *mirroring* or *re-enacting* their mental states and physical activities (Blakemore and Decety, 2001; Rizzolatti et al., 2006).

The present paper focuses on action simulation. It aims to discuss theoretical and experimental approaches to action simulation and its cognitive underpinnings, broadly understood as internal representations that parallel external action events, like observing a friend making a cup of coffee or a couple dancing together. From a functional point of view, internal simulations of physical actions may improve our appraisal of actions that we plan to perform in collaboration with others and that require us to act in response to and in anticipation of the actions of others (e.g., Sebanz and Knoblich, 2009; Doerrfeld et al., 2012; Manera et al., 2013). A recent study illustrative of this demonstrated that judgments of the weight of an object varied according to whether the participants planned to lift the object by themselves or whether they planned to lift it together with a co-actor who was either healthy or injured (Doerrfeld et al., 2012).

Here, we use the term simulation to refer to the mental operations involved in internally representing actions during so-called visual action occlusions. In everyday life, when we watch other people moving around us, it often happens that they disappear from sight for a moment. However, in these situations, behaviours and physical actions observed immediately prior to occlusion do not just stop. Based on what we have seen before, we are usually capable of internally substituting (simulating) the invisible parts of an action and rendering quite precise predictions about its future course (i.e., what the agent will do, and when his/her action will take place).

Neurophysiological evidence in monkeys demonstrates that neurons continue to fire for some time in response to specific actions even after these actions disappear behind an occluding object (Umilta et al., 2001; Jellema and Perrett, 2003). In human experiments using action occlusion paradigms, participants typically watch familiar actions that are briefly occluded from view and then continued. A typical task is then for participants to indicate if the re-appearing action part (after occlusion) is an accurate continuation of the perceived action (seen prior to occlusion), or if it has changed in spatial angle (e.g., Graf et al., 2007; Springer et al., 2011) or has jumped in time (e.g., Stadler et al., 2012b). High accuracy on this task requires a precise internal representation (simulation) of the occluded action part, and factors that influence performance can then be measured. For instance, Stadler et al. (2012b) examined the accuracy of action simulation when the observed actors moved according to human-like vs. artificial motion profiles whose kinematics had been changed according to a non-human, constant velocity. Under conditions with temporary occlusions, observers were clearly more able to predict human-like actions compared to non-human actions (Stadler et al., 2012b), highlighting the fundamental susceptibility of the human action simulation system (Parkinson et al., 2012; Saygin and Stadler, 2012; see Gowen and Poliakoff, 2012, for a review).

Since temporary occlusions require switching back and forth between externally guided perception and internal representations (simulations), action occlusion paradigms, further, allow the study of these two phases separately and in terms of their interrelations. In an illustrative study by Prinz and Rapinett (2008), the participants observed a human hand transporting an object. After a moment, the hand with the object was briefly occluded from view. Participants were required to make a judgment about the time that they thought the transporting hand with the object would reappear from behind the occluding object (Figure 5). Results indicated a substantial positive time error (i.e., lag effect), meaning that the continuation of the action after occlusion was judged to be “just in time” when the point of reappearance was slightly shifted ahead (by 20–100 ms). This finding provided a first insight into the timing and nature of internal action representations (simulations) during occlusions, suggesting that the mental operations called up during occlusions involve the generation of novel action representations rather than just pure extrapolation of perceived movement trajectories (Prinz and Rapinett, 2008).

The authors of this study put forward that these issues are based on the assumption that unlike action perception, which naturally draws on external resources derived from actual stimulation, action simulation draws on internal resources derived from stored knowledge (cf. Prinz and Rapinett, 2008). In the following section, we focus on the temporal characteristics of action simulation. Based on the experimental evidence from different occlusion tasks, we propose that action simulation engages internal online processes operating in real-time, which act on newly created action representations rather than relying on continuous visual extrapolations of observed movement trajectories.

In Section Representational Mechanisms, we discuss another strand of experimental studies that have explored the procedural and representational characteristics of the processes engaged for solving action occlusion tasks. Two major findings emerge. Firstly, predicting occluded actions may engage two distinct processes: dynamic simulation and static matching. Both processes do not by themselves speak to the representational format in which they occur (e.g., simulation/matching in the motor and/or visual domain). Secondly, two different kinds of representational domains may be involved: sensorimotor processes (those involved in an observers' own physical activity) and semantic processes (those involved in understanding action-related verbal contents). In a concluding section, we propose that the concept of internal action simulation can be related to a predictive coding account of motor control (e.g., Kilner et al., 2007), in correspondence with the broader notion that humans can use their motor system to simulate and predict others' actions (Grèzes and Decety, 2001; Jeannerod, 2001). In line with this notion, action simulation may also be linked to embodied views of language, holding that processing verbal and conceptual action-related information is strongly linked to (and may even rely on) information processing in sensory and motor domains (e.g., Barsalou, 2003, 2008; Zwaan, 2004; Pulvermüller, 2005, 2008; Glenberg, 2008; Mahon and Caramazza, 2008, 2009).

## Time course

### Simulation in real time: the occluder paradigm

The first research into real-time action simulation used what we refer to as the *occluder paradigm*, first developed by Graf et al. (2007). This paradigm has been used, with some novel variations, in subsequent research on action simulation (Prinz and Rapinett, 2008; Parkinson et al., 2011; Sparenberg et al., 2012). The occluder paradigm is based on the hypothesis that when we observe a human moving, who is then occluded from view—perhaps he or she disappears behind a fence—an internal action simulation runs in real-time predicting the on-going motion. Then the person reappears in view at a point in the motion that either matches or does not match that internal real-time simulation. In this way, the spatiotemporal accuracy of the action simulation and the ability of people to use action simulation to aid their perception and prediction of human movement can be tested.

**Figure 1:**
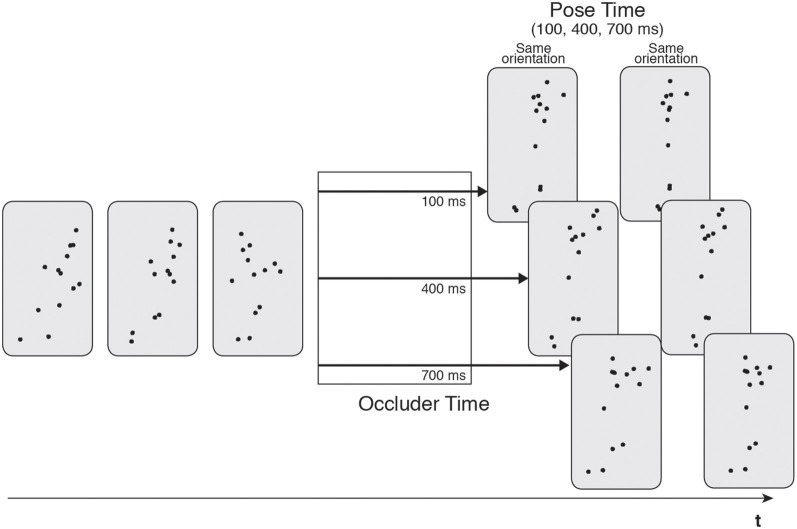
**Illustration of the stimuli utilized in Graf et al. (2007)**. Point light actions were presented and then occluded for a variable time (100, 400, or 700 ms). Occlusion was followed by a test pose that was rotated or in the correct orientation. Pose time was also varied (100, 400, or 700 ms).

The original occluder paradigm (Graf et al., 2007) used representations of human motion known as *point-light actors* (PLAs; Johansson, 1973), which convey motion via a group of moving dots that track the motion of the major joints of the human body. These stimuli have been widely used to examine human movement processing (Johansson, 1973, 1976; Cutting et al., 1988). Graf et al.'s (2007) original studies used PLA stimuli of non-cyclical human motions, such as performing a basketball shot or hitting a tennis ball. The PLA was presented to the participant for a short period of 2–4 s before being occluded from view for a fixed amount of time (occluder time; 100, 400 or 700 ms, see Figure 1). Following this, a static test posture of the action was presented that was either rotated around the actor's vertical axis, as if he had suddenly spun to the left or the right, or in the correct orientation, as if he had continued smoothly on in his motion. The participants' task was to judge whether this rotation had occurred or not (yes/no response). Crucially, and independent of the spatial orientation, the test posture was either a congruent or incongruent continuation of the motion. That is, if the test posture was congruent, it was taken from the point in the motion that the actor would have reached if he had continued for the exact duration of the occluder. In this condition, the test posture should match the state of the internal real-time simulation. In the incongruent conditions, the test posture was from a point of the motion that was too early or too late with respect to the exact occlusion period. In this case, the test posture would not match the current state of the real-time action simulation. The occlusion period is referred to as the *pose time* (which, again, can take three values, 100, 400 or 700 ms, see Figure 1).

The hypothesis was that if the test posture matched the current state of the action simulation, then the orientation judgment would be easier and more successful. This was indeed the case: error rates for congruent continuations were less than those for incongruent continuations across different occluded durations, a finding that was demonstrated across a number of different types of human motions. This was the first evidence that the real-time action simulation existed and that it could be tested by utilizing its beneficial effects in visual judgment tasks. Graf et al. (2007) conducted a further experiment using PLAs that were inverted on their vertical axis; it is well-known that it is difficult to perceive human motion under these conditions. The results showed that there was no judgment-benefit for the congruent test postures compared to the incongruent ones. This suggested that the effects seen in the earlier studies were specific to human motion itself and not some more generalized visual predictive system. That is, the real-time simulation is specifically concerned with predicting coherent examples of human actions.

### Benefits of real-time simulation

Having described the occluder paradigm for testing action simulation, we will now review recent research that has used and further developed it to measure various aspects of action simulation, such as its precise time course, its susceptibility to online change, and its role in the direct visual perception of human motion.

#### Detection thresholds

First, we consider the advantageous role that real-time action simulation has in the visual processing of human motion. That is, can we show that the action simulation can provide a here-and-now, or predictive, benefit to visual perception? Imagine watching a football match on television. The TV is an old analogue set and is fed by a radio antenna on the roof. It's windy, the antenna is blown around, and the TV picture is occasionally replaced with “snow”: the random cascade of black and white dots that represent a lack of signal. A player is about to score when he disappears briefly under this visual snow and you are trying to keep track of him until he reappears on the screen in the haze of bad picture quality.

We argue that this is when action simulation might occur: you generate a real-time model of a player's movements whilst he is briefly occluded from view. This example also illustrates what may be an adaptive benefit of the real-time action simulation: if the internal model tracks the exact time-course of the player's movements, the model can then be used to provide a perceptual prediction—also in real-time—of how the player should look at any moment during occlusion. If the figure is not fully occluded but simply visually degraded, the perceptual prediction may provide real-time support to the visual system, aiding the detection and processing of the faintly seen player. Importantly, if the faintly reappearing player was not then moving in a way consistent with the real-time predictive simulation—the video had skipped forward or backwards, for example—the simulation would provide no such predictive benefit.

**Figure 2:**
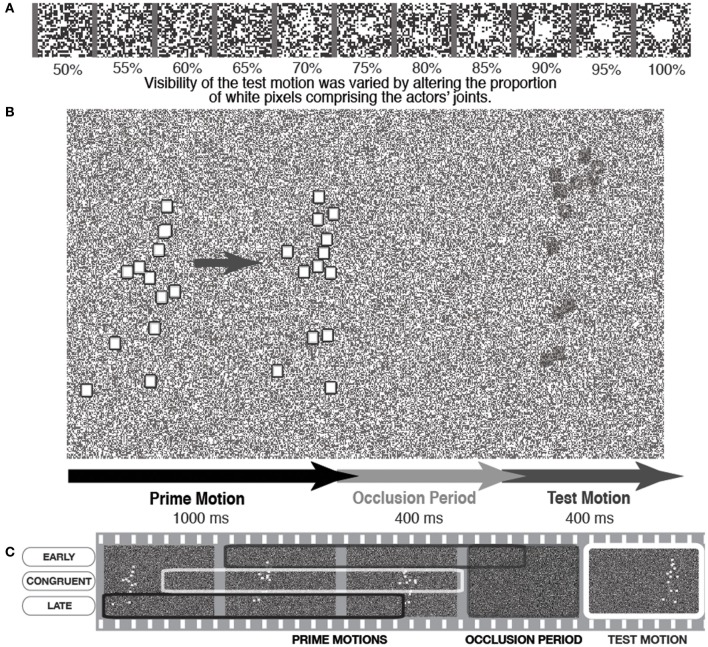
**A schematic illustration of a trial in which the PLA was presented with variable visibility against the background. (A)** Shows how varying white pixel ratio in the actor's joints increases visibility against the noise background, **(B)** shows a basic trial sequence, and **(C)** depicts a schematic showing how different sections of the action were shown as the prime motion to manipulate motion congruency with the same test motion section. Figure adapted from Parkinson et al. (2011, p. 1466). Copyright © The Experimental Psychology Society. Adapted with permission of Taylor and Francis Ltd., www.tandfonline.com on behalf of The Experimental Psychology Society, with permission from the authors.

This was the rationale behind a recent set of experiments (Parkinson et al., 2011) in which PLAs were presented on top of a constantly changing random pattern of black and white pixels in a 50/50 ratio, resembling TV “noise.” The points of the actor were squares of pixels that could also be rendered as randomized patterns of black and white dots, with a variable ratio of white dots to black dots. If the ratio of white dots was 100%, the actor was clearly visible. However, as the white ratio was reduced the actor was less visible against the background noise, until a 50% ratio rendered him totally invisible (see Figure 2A).

In each trial, the participants were presented with background noise that continuously changed throughout the trials. Participants clearly saw the initial part of the action, which we refer to as the *prime motion*, as it was the section of motion that was assumed to prime the generation of the subsequent action simulation. Then the actor was occluded from view for a short period (400 ms) after which he reappeared, in motion, for 400 ms. This was the *test motion* that was presented with variable visibility against the background (see Figure 2B). The participants' task was to indicate whether they saw the reappearing actor or not, with test motion visibility adaptively altered to reach set detection rate targets. Thus, detection thresholds for the test motion could be measured in terms of the ratio of white pixels depicting the test motion actor. This was an indicator of how easy participants found it to detect the reappearing actor under difficult visual conditions.

It is important to note that this detection task is a simple, immediate “here-and-now” judgment, as opposed to the more postdictive one used in the original Graf et al. (2007) paradigm. That is, participants were not asked to make any judgment about the quality of the reappearing actor (e.g., whether he had turned or not), but quite simply whether he had reappeared at all. Thus, this paradigm made it possible to measure whether action simulation aided the basic visual prediction and subsequent detection of human motion (i.e., detection thresholds). An action simulation would be generated during the occlusion, which was a continuation of the prime motion seen prior to occlusion. In order to test the simulation's effect on test motion detection, the spatiotemporal relationship between the prime motion and the test motion was manipulated. This meant that the action simulation would be congruent with the test motion, or the test motion would be “too early” or “too late” to match the action simulation (incongruent conditions). To avoid confounds of test motion on detection thresholds, the same section of test motion was used in all congruent and incongruent conditions. Thus, the spatiotemporal manipulations were achieved by presenting different sections of prime motion, which would subsequently drive different action simulations in relation to the single test motion (see Figure 2C).

Detection thresholds were measured for a variety of actions in three conditions: action simulation-congruent test motions, incongruent early test motions, or incongruent late test motions. Congruent thresholds were consistently lower than those for either of the incongruent condition. Hence, if the currently generated action simulation was temporally congruent with the test motion, the latter was more easily detected. These experiments suggest that the action simulation can have a direct, immediate, here-and-now benefit for the perception of human movement, but only when the external stimulus of movement temporally matches the internal action simulation. This suggests some form of on-going, real-time, top-down effect of action simulation on ongoing visual processes, and supports the notion that internal forward models for biological motion and action can be used to directly supplement the perception of those actions (Wilson and Knoblich, 2005; Prinz, 2006).

#### Inserted motion

Another consideration in understanding action simulation is how stable the internal real-time model of motion is. In other words, is it possible to briefly bias, or indeed replace, the current ongoing action simulation by very briefly introducing human motion information that does not match the current state of the ongoing simulation process? Parkinson et al. (2012; Experiment 2) investigated this question by adding “inserted motion” in the occluder. Specifically, PLAs were presented that were briefly occluded for 500 ms and then reappeared in motion for 500 ms (i.e., test motions). These test motions were either temporally congruent with the ongoing action simulation at that point or were temporally incongruent, that is, offset 267 ms too early or too late in the motion sequences. The participants were asked to make an explicit 2-alternative forced-choice judgment as to whether the test motion was a correct continuation or not, that is, the judgment measured how well the test motion matched the action simulation that was being generated at that point in time.

**Figure 3:**
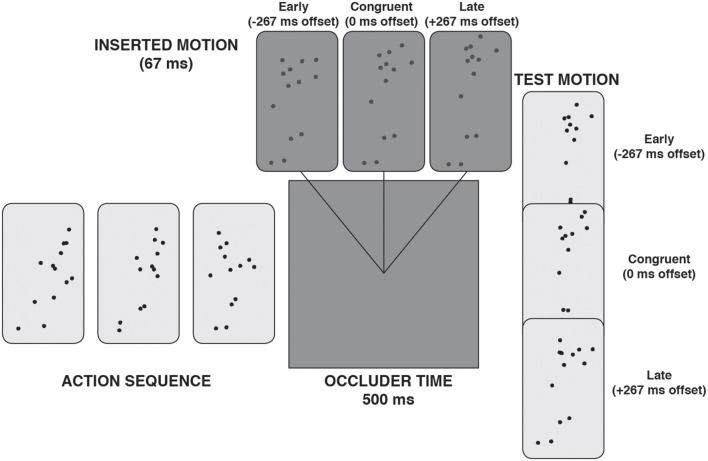
**A schematic illustration of a trial with “inserted motion” during the occlusion phase**.

Around the temporal halfway point of the occlusion period, 67 ms (4 frames) of low contrast PLA was presented, such that participants got the impression of a brief “flash” of motion within the occlusion period (see Figure 3). Crucially, the inserted motion was either temporally congruent with the action simulation or too early or too late by 267 ms. In the congruent instance, the inserted motion matched the action simulation, almost as if the ongoing action was seen very briefly through a slit in the occlusion period. In the two incongruent instances, the inserted motion would not match the ongoing action simulation. The hypothesis was that, despite the brevity of the inserted motion, it would nevertheless act to bias or replace the action simulation.

**Figure 4:**
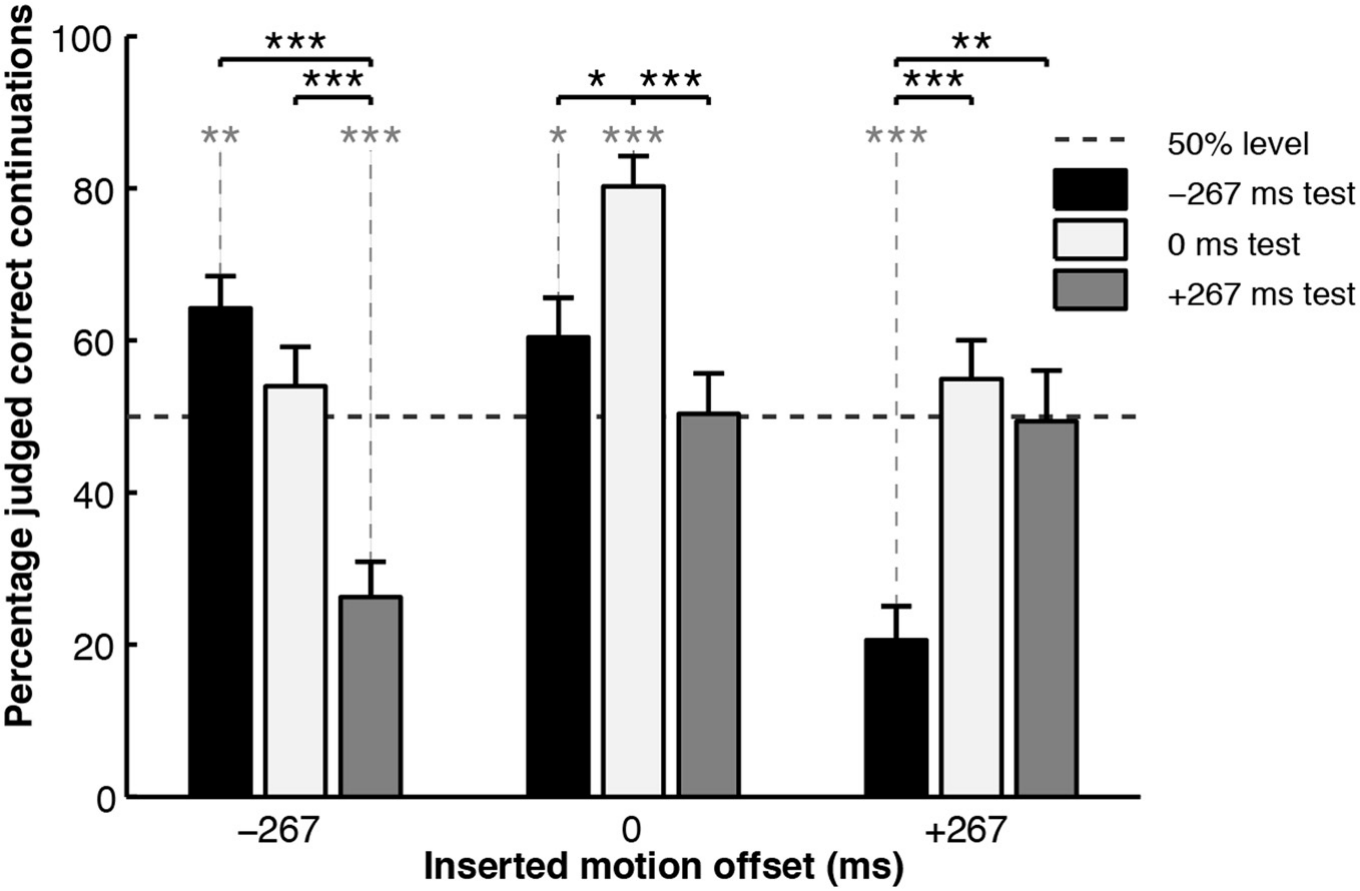
**Percentage correct judgments for the “inserted motion” experiment**. Black asterisks connected with solid lines indicate significance levels of between-condition *t*-test comparisons. Asterisks in gray connected with dotted lines indicate significance levels of one-sample *t*-test comparisons to chance performance (50%), ^*^p < 0.05, ^**^p < 0.01, ^***^p < 0.001. Figure reproduced from Parkinson et al. (2012, p. 428). Copyright © Springer Science+Business Media. Reproduced with permission.

The experiment, thus, represented a 3 (test motion offset) × 3 (inserted motion offset) design, with the measurement being the percentage of trials in which the participants judged the test motion to be a “correct continuation.” The results are shown in Figure 4. When the inserted motion matched the action simulation (0 ms offset, central cluster), it did not interfere with the action simulation process and the results were as expected: participants were more likely to judge the congruent test motion as correct, as opposed to either of the incongruent test motions, showing that they could utilize the action simulation to correctly judge the veracity of the test motion. However, the pattern of results was distinctly different when the inserted motion was incongruent with the action simulation: when the inserted motion was offset in one temporal direction, judgments of what was a correct test motion also shifted in that temporal direction.

This suggests that the action simulation process can be updated “mid-flow” by new incoming motion information, no matter how briefly that new motion is perceived for. This is perhaps unsurprising because an action simulation process that remains immune to change may not be a very useful mechanism for predicting the ongoing movements of others. Two possibilities arise as to how the inserted motion effects this updating of the action simulation: firstly, the *biasing hypothesis* suggests that briefly presented motion that does not temporally match the current state of the action simulation acts to fluidly update it, temporally “pushing” the action simulation in that direction. The second hypothesis suggests that *re-simulation* occurs: perceiving even a short duration of inserted motion cancels the currently generated action simulation and generates a new one based on the new motion information. Naturally, if the inserted motion matches the old simulation, the new one will roughly match the old one, leading to the expected results, as seen in the 0 ms inserted offset condition described above. However, if the re-simulation is based upon temporally shifted motion, the newly generated simulation will perceptually support “incorrect” continuation test motions.

Both of these hypotheses are possible and the current evidence does not conclusively support one or the other (Parkinson et al., 2012). It might seem, on the face of it, that the re-simulation hypothesis is less intuitively sensible, since it would entail that an entirely new action simulation can be accurately generated from only 67 ms of a PLA motion. In fact, this is entirely feasible, as we will see later (Section The Lag Effect: Towards a Higher Temporal Resolution).

### The lag effect: towards a higher temporal resolution

We have described recent research, which, in the first instance, shows the existence of real-time action simulation of human motion (Graf et al., 2007; Parkinson et al., 2011). Secondly, we have demonstrated an ecologically valid real-world benefit of action simulation, which can act in a top-down fashion to aid human motion detection (Parkinson et al., 2011). Finally, we went on to illustrate the fluid, updatable nature of the simulation (Parkinson et al., 2012). Now, we turn to the investigation of the spatiotemporal accuracy of the action simulation: the more fine-grained nature of how well the simulation can track human motion.

#### Spatial occlusion and the teapot experiment

**Figure 5:**
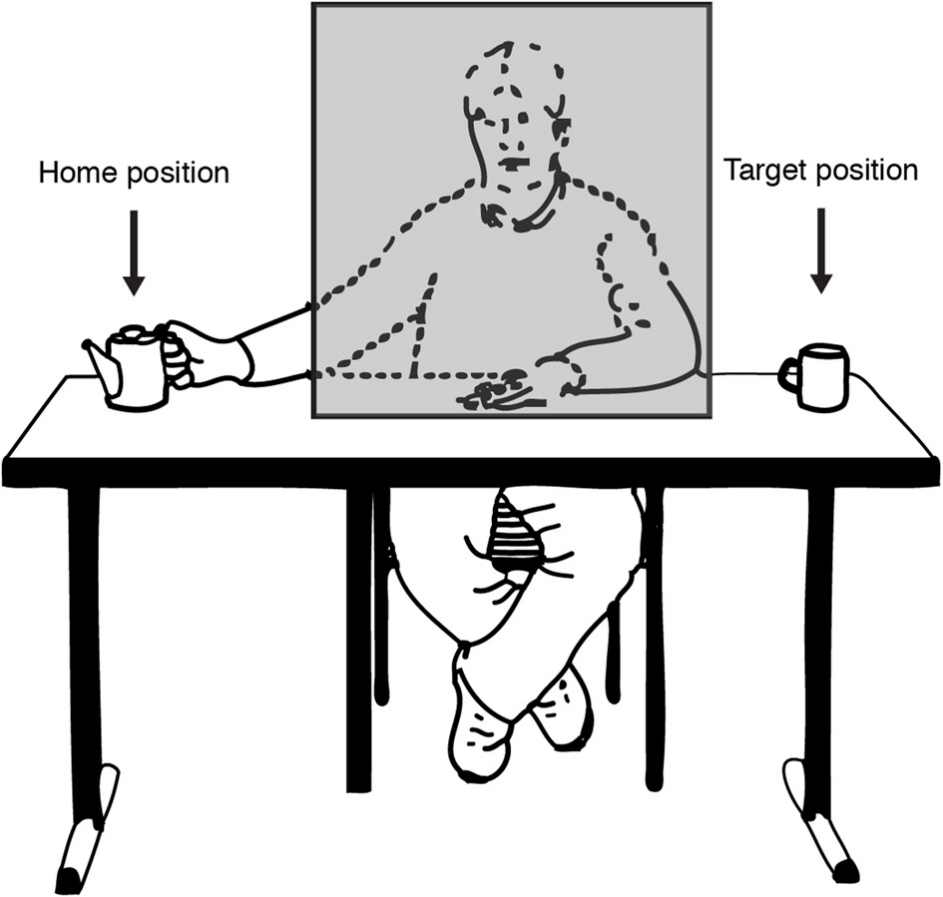
**Illustration of the experimental setting as seen through the eyes of the participant**. On each trial the actor (sitting behind an occluding object) transported a teapot from a home position to a target position. Figure adapted from Prinz and Rapinett (2008) (p. 226). Copyright by IOS Press. Adapted with permission.

Work by Prinz and Rapinett (2008) attempted to investigate the time-course and accuracy of action simulation by asking how it relates, at first, to simple visual linear extrapolation. They used a novel version of the occluder paradigm, which used actual video footage of people making simple goal-directed motions. The actors of these videos sat facing the viewer but hidden behind a permanently present cardboard occluder (see Figure 5). The motions they performed were simple, manual transport movements, such as reaching with their right hand for a teapot on their right (screen-left), picking it up, and moving it screen-right behind the occluder, to reappear screen-right of the occluder where a mug or cup awaited the teapot. Since the occluder was onscreen throughout, the moments and position of the start of the occlusion were highly predictable, as was the spatial position of reappearance considering the linear left-right trajectory of the transport motion. Therefore, the experiment was ideally suited to measure the spatiotemporal accuracy of the action simulation by examining participants' judgments of the time the teapot reappeared from the right-edge of the occluder.

**Figure 6:**
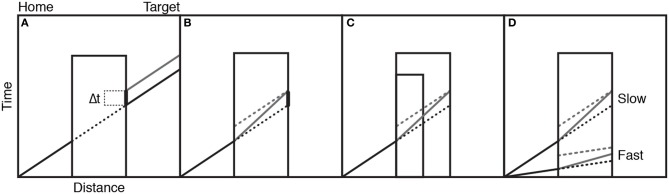
Panel **(A)** shows the actual movement of an object behind the occluder (black lines) and the action simulation (gray line) illustrating lag error. Panel **(B)** shows two sources of the lag error: intercept (dotted gray) and slope (solid gray) lines. Panel **(C)** shows the different predictions of the two sources of lag error when occluder duration changes. Panel **(D)** shows the different predictions of the two sources of lag error when motion speed changes. See text for detailed explanations. Figure adapted from Prinz and Rapinett (2008) (p. 226). Copyright by IOS Press. Adapted with permission.

The teapot could reappear either at the correct time, as if the motion had continued as normal behind the occluder, or too late or too early in steps of 40 ms. Participants judged whether the teapot appeared too late, just in time, or too early. When the “just in time” judgments were analyzed, it was found that there was a positive time error in the judgments. That is, the reappearances participants thought were correct were, in fact, too late. This suggests that the action simulation is not entirely accurate in tracking ongoing occluded motion; there is some temporal lag present. Figure 6A schematically illustrates these results on the assumption that the transport motion (solid black line) has a constant velocity as the teapot is moved from screen left to right. The black dotted line represents the occluded portion of the motion. This would also represent the trajectory of an accurate action simulation: one without lag. The gray line represents the perceived trajectory of the teapot after occlusion, with a positive time lag, which equates with the time lag in the “just in time” judgments.

Retaining the assumption that an action simulation has a linear velocity profile like the action it represents, there are two possible sources for the judgment error. Firstly, the generated action simulation may be slower than the actual action. This is called the *slope error*, and is represented in Figure 6B as the solid gray line within the occluder. The second source of error comes from instances where the action simulation matches the real action in terms of speed, but there is a time-cost involved in generating an action simulation, which means it lags behind the action by a set amount from the start. This is represented as the dotted line in the occluder in Figure 6B, and is known as the *intercept error*. The two errors are not mutually exclusive.

In order to ascertain which of the two errors contributes to the lag in continuation judgments described above Prinz and Rapinett (2008), conducted a second experiment in which two different sizes of occluder and two different speeds of motion were used. Altering the occluder size changes both the distance over which the action is occluded and the time taken until reappearance (Figure 6C). Altering the speed of the motion alters the amount of time it takes the motion to cross the same occluder distance (Figure 6D). In both cases the slope and intercept error hypotheses make markedly different predictions. The simulations affected by intercept errors are shown in dotted gray lines, and those affected by the slope error are in solid gray. Because the time cost involved will occur from the start of simulation, this lag should be constant, irrespective of the occluder size, and so judgment lags will remain constant across occluder conditions. However, the slope error hypothesis implies that the longer the action is occluded for, the more the lag increases. Hence, a larger occluder should produce more error than a smaller occluder (Figure 6C). Similarly, increasing the speed of the action and, thus, decreasing occlusion time should, according to the slope hypothesis, decrease lag error, whilst again the intercept error suggests that lag will be the same irrespective of action speed (Figure 6D).

However, when an experiment was run combining two transport speeds with two occluder widths, the results were the opposite of the predictions of both the slope and the intercept error hypotheses, with lag error being smaller for slower action speeds, and smaller for longer occluders. This means, firstly that the constant cost (intercept) hypothesis must be rejected, because it predicted that error would be constant. Interestingly, however, it also suggests that the source of the lag error cannot be a slowing of a linear extrapolation (slope error) because the results are the opposite of what that hypothesis predicts.

**Figure 7:**
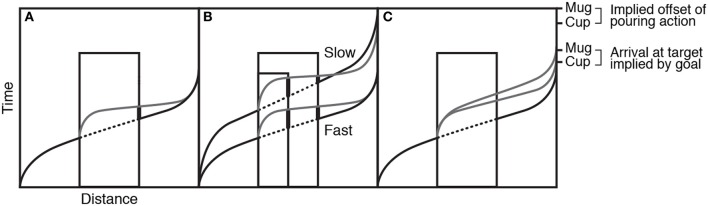
Panel **(A)** shows the velocity profile of the action as it accelerates from the start and decelerates at the target (black solid line) with the occluded portion dotted. The regenerated action simulation is shown in gray. Panel **(B)** shows how this regenerated simulation hypothesis provides different predictions when occluder duration and action speed change. Panel **(C)** shows how action simulations might be affected by the implied goal of the action. Figure adapted from Prinz and Rapinett (2008) (p. 226). Copyright by IOS Press. Adapted with permission.

Since the available evidence did not support action simulation as a simple, linear extrapolation of the occluded motion, Prinz and Rapinett (2008) went on to reconsider the nature of the simulation: First, they included more details about the spatiotemporal properties of goal-directed movements, namely that a goal-directed transport movement tends to have a period of acceleration at the start and a deceleration toward the goal at the end. Second, they suggested that rather than being a simple extrapolation or continuation of the movement, the action simulation is actually an internally generated re-start of the motion. As the visual input of the goal-directed input is removed at the occluded edge, the action simulation may generate a model of a similar goal-directed action with the same target (end-point) but with a new start point that of the occluded edge. This means that the action simulation entails a period of acceleration from its own start, then moves and decelerates toward the exact same spatiotemporal target of the original action. Figure 7A shows the velocity profile of the action as it accelerates from the start and decelerates at the target (black solid line) with the occluded portion dotted. The re-generated action simulation is shown in gray, with a similar accelerating-decelerating profile. The thick line on the right side of the occluder highlights the magnitude of the lag error. Figure 7B shows how this re-generated simulation hypothesis can account for the previously puzzling results: faster actions produce more lag error than slower actions and larger occluders produce more error than smaller occluders.

In a final experiment, Prinz and Rapinett (2008) looked at the effects of implied goal duration and produced a remarkably effective demonstration that action simulation involves the internal modeling of goal-oriented human action and not merely visual prediction of kinematics: they used the same video-based paradigm involving a left–right teapot transport with two different occluder widths. In addition, they varied the visual identity of the target item between a small cup and a large mug. The large mug would take longer to fill than the small cup, so the length of time taken to achieve the action-goal should be longer for the mug than the cup. While the videos of the reappearing movement always stopped at the same point, just before the contents of the teapot were about to be poured, a greater positive lag error was observed in response to the mug compared to the cup targets, meaning that the greater amount of time implied for filling the mug had increased the target time for the generated action simulation (see Figure 7C).

This work by Prinz and Rapinett (2008) simply, but effectively, demonstrates a number of details regarding both the generation and the spatiotemporal details of action simulation. Firstly, the simulation is not merely a linear extrapolation or continuation of the perceptual information; indeed, it seems not to be a continuation at all. Instead, it may actually be that an entirely new model of the goal-directed action that has been occluded is generated, but starting from the point of occlusion, and this re-generation utilizes goal-directed kinematic information inherent in action systems. In this sense, the re-generation may, in fact, be more closely tied to motor systems than perceptual systems, in that it uses goal-directed motor information to supply the perceptual information, a notion put forward by Prinz (2006) and Wilson and Knoblich (2005).

Sparenberg et al. (2012) took a more detailed look at the lag error in action simulation measured by Prinz and Rapinett (2008). They used PLA stimuli and a 300 ms occluder period, after which they showed a static test posture, which could be offset earlier or later than the true posture of the actor immediately following occlusion. Participants were asked if the test posture was too late or too early to be the correct continuation of the motion. Results showed that test postures that were too early in the sequence were judged to be a correct continuation. That is, over a fixed period of occluder time, the action simulation lags behind the true motion. When Sparenberg et al. (2012) measured this lag over two different occluder durations, they found that the lag did not change but remained constant at 25 ms lag error. This contradicted the findings of Prinz and Rapinett (2008) that the error reduced with longer occlusion durations (and also movement speed, not manipulated by Sparenberg et al., 2012), and varied between 18 and 141 ms. Sparenberg et al. (2012) concluded that the stable lag error was a result of a constant time-cost when switching from perception of motion to internal action simulation.

It should be noted that, on closer inspection, it is very difficult to directly compare the two paradigms. For instance, in the stimuli used by Prinz and Rapinett (2008), the occluder was permanently visible, meaning that the point of occlusion was spatially and temporally predictable, and the point of reappearance was at least spatially predictable. In comparison, in Sparenberg et al. (2012) the “occluder square” would suddenly appear and then disappear on the screen, meaning that the spatiotemporal point of occlusion was less predictable, and the position of the reappearing test posture was also not predicted by any properties of the occluder. Secondly, the nature and complexity of the motions that were to be simulated differed greatly between paradigms: the Prinz and Rapinett (2008) transport movement is much more linear in nature than the full body motions used by Sparenberg et al. (2012). This action also only uses a single limb and the individual arm movement has a clearly defined (or implied) end-point or goal.

The combination of predictable occluder onset and offset, plus the simpler, linear quality of the motion in the Prinz and Rapinett (2008) paradigm may contribute to a greater ability to simulate a goal-directed end-point for those actions and thus produce an action simulation with the spatiotemporal properties shown in Figure 7. In this situation, lag error will vary according to occluder duration and action speed, as previously discussed (Figure 7B). On the other hand, it may not be possible to generate a simulation based on a goal-directed end-point for more complex full body motions, as used in Sparenberg et al. (2012). In this case, the action simulation may predict the ongoing complex motion in a more linear fashion, meaning constant lag costs irrespective of occluder duration. Further experiments are needed to tackle this issue.

Still, the findings from both paradigms are informative regarding the nature of action simulation and its underlying dynamic processes, suggesting that, whilst the precise temporal nature of the simulation may vary with the type of action being simulated, the existence of temporal lag is common.

#### Motion information required for action simulation generation

As described earlier, Prinz and Rapinett (2008) suggested that action simulation is not merely a perceptual–continuation mechanism but is instead a generative internal modeling that uses information about the perceived motion—and perhaps also motoric knowledge regarding that action—to produce a new goal-directed simulation of the action. If the simulation is generated using motor as well as perceptual information, a pertinent question to ask is: exactly how much visual motion information is required to generate the action simulation?

To test this issue, Parkinson et al. (2012; Experiment 1) used a PLA version of the paradigm, in which the initial prime motion of the PLA was occluded for 500 ms followed by 500 ms of the reappearing actor in motion. The crucial manipulation was the duration of the pre-occluder motion: with each frame of the PLA animation lasting 10 ms, the prime duration was varied to be either 20, 50, 100, 500, or 1000 ms (i.e., the last condition only presented 2 frames of PLA motion before the occluder). Different sections of test motion were presented in method of constant stimulus (MOCS) fashion and participants were asked to judge if the test motion was too early or too late to be a correct continuation. This allowed the accuracy of the action simulations in terms of its temporal lag to be computed in a similar way to that used by Prinz and Rapinett (2008).

**Figure 8:**
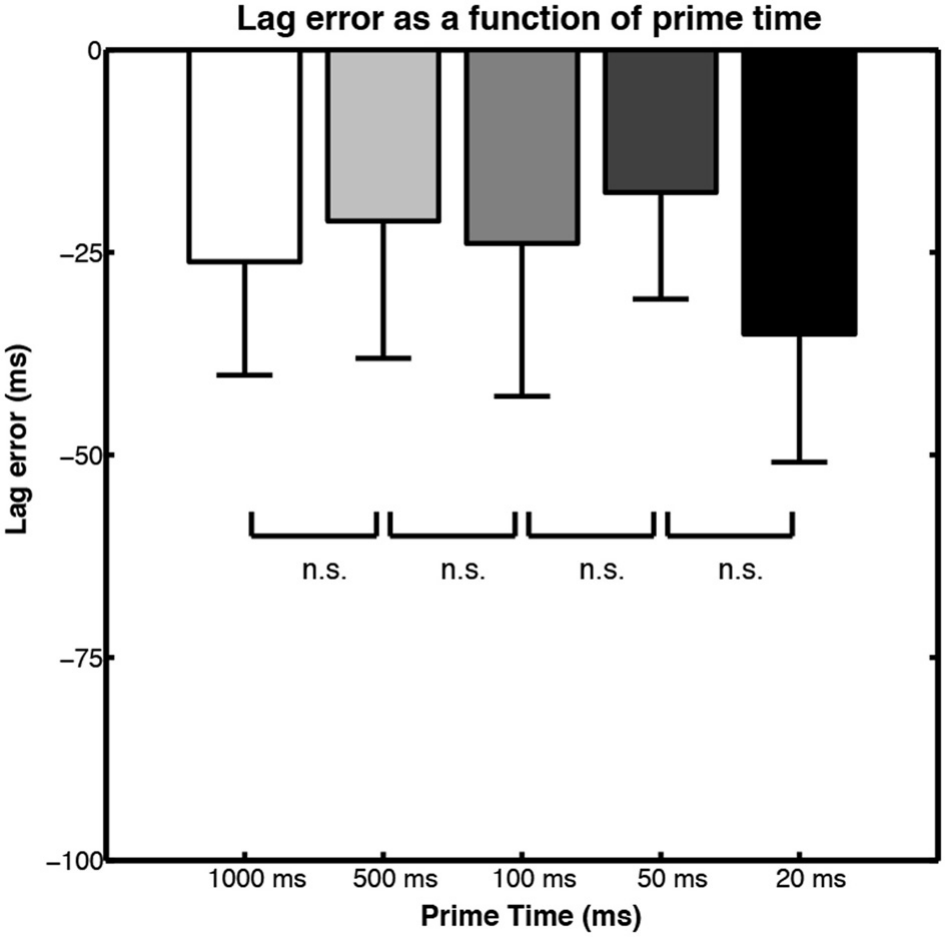
**Bar graphs of lag error in action simulation motion judgments when different durations of motion are shown prior to occlusion**. Figure adapted from Parkinson et al. (2012, p. 426). Copyright © Springer Science + Business Media. Adapted with permission.

The mean lag errors for each of the prime motion durations are shown in Figure 8. The lag errors are all negative, meaning that participants tended to judge early offset test motions as being correct continuations. This implies that the action simulation is running slightly slower than the action it is being generated to predict. This is a result we have already encountered in Section The Lag Effect: Towards A Higher Temporal Resolution where the Prinz and Rapinett (2008) and Sparenberg et al. (2012) studies are detailed. What is remarkable in Parkinson et al. (2012) experiment is that the temporal accuracy of the action simulation was not affected by the amount of human motion provided before the occluder: even when presented with as little as 20 or 50 ms of PLA motion (2 or 5 frames), the generated action simulation was just as temporally accurate as it was when participants saw 1 s of motion before the occluder.

Of course, during the course of an experiment the actions will become familiar, so generating a simulation from a brief glimpse of a PLA may not be a finding that will generalize to other situations, but it is still an interesting finding. This relates to Section Benefits of Real-Time Simulation, in which we described how inserting a very brief amount of point light motion within the occluder can bias subsequent judgments of reappearing motion (Parkinson et al., 2011). We suggested two mechanisms for this: 1) that the inserted motion biases the currently generated action simulation, or 2) that the inserted motion is used as the basis for a re-simulation and the generation of an entirely new action simulation based on the new motion percept. At first the re-simulation notion seems less appealing: is 67 ms of human motion enough to generate a whole new predictive model?

However, the results of the experiment by Parkinson et al. (2012) point to just this and it had been shown previously that very brief exposures to biological motion can provide enough information for adequate processing (Thirkettle et al., 2009). The results of Parkinson et al. (2012) suggest that the action simulation is remarkable in that it can generate real-time motion predictions from very short exposure to familiar human movements, and this perhaps accounts for the results when brief conflicting motion information is inserted in the occluder: the action simulation is re-started using the new motion section. The notion of re-simulation is also appealing in light of the hypothesis brought forward by Prinz and Rapinett (2008), namely that action simulation involves the generation of an internal forward model that combines current motion information, motor knowledge, and information about the implied end-point of the motion.

### An interim summary

Action simulation is a process that internally models human movements in real-time. The process of action simulation can be demonstrated and investigated using the occluder paradigm (Graf et al., 2007; Prinz and Rapinett, 2008), in which a human actor disappears from view and then reappears at a position/motion–continuation which can be either correct—as if they had continued moving behind the occluder—or from too late or too early in the sequence—as if the “video” of the motion skipped forward or back. When participants are tested on some orthogonal aspect of the reappearing actor, for example when asked “Has her form been rotated?”, they perform better when the test position of the actor is correct with respect to the length of the occlusion. This demonstrates that the action simulation is real-time in nature, modeling the position of the occluded actor at that temporal point (cf. Section Simulation in Real Time: The Occluder Paradigm).

Action simulation has been demonstrated to directly aid the visual perception of a visually degraded human motion, but only when that motion spatiotemporally matches the real-time state of the action simulation (Sections Benefits of Real-Time Simulation and Detection thresholds). We have detailed how visual exposure to even very short durations of human motion can provide sufficient information to generate an action simulation (Sections The Lag Effect: Towards A Higher Temporal Resolution and Motion information required for action simulation generation). We have also described the way in which the ongoing time-course of the action simulation can be manipulated by displaying very short sections of the motion during occlusion, which could again be either temporally congruent with—or earlier or later than—the real-time state of the action simulation at that point. These tended to bias judgments of which reappearing motion was a “correct continuation” in the temporal direction of the inserted motion (Section Benefits of Real-Time Simulation, “Inserted motion”). This illustrates that the action simulation can be updated in real-time.

Finally, whilst it is clear that the action simulation is real-time, in that it unfolds over time as the real action does, the simulation slightly lags the action (Section The Lag Effect: Towards A Higher Temporal Resolution, “Spatial Occlusion and the Teapot Experiment”). Research into the source of this lag error has suggested that the simulation itself is not simply a linear extrapolation of the visual motion of the action before occlusion. Instead, the process of action simulation involves an internal generation of a model of the movement that includes the velocity and acceleration profiles of a newly initiated goal-directed action. This model uses the spatiotemporal point of occlusion as the starting point and the implied goal of the action as its end point. Taken together, we see that action simulation is a process which generates a real-time model of an action that takes into account the goals of the action, probably using one's own implicit motor knowledge, and that the action simulation can be dynamically updated and provide direct perceptual benefits when a human motion is difficult to see.

## Representational mechanisms

While the studies discussed above indicated that perceptual processes can strongly impact how we perceive and predict others' actions, a number of significant unexplored questions regarding the role of motor processes remain. In the following, we discuss this issue based on studies examining how an observer's own physical activity may affect his or her ability to accurately simulate and predict others' actions.

### Sensorimotor processes

A wealth of experimental research has demonstrated strong mutual influences between action perception and execution (for a review see Schütz-Bosbach and Prinz, 2007). While motion detection is impaired when the motions go in the same direction as concurrently performed actions (Hamilton et al., 2004; Zwickel et al., 2007), movement execution depends on similarity-based relationships between go-signals and movements to be performed (Brass et al., 2001; Craighero et al., 2002) and exhibits greater variability when a different movement is concurrently observed (Kilner et al., 2003).

Recent experiments have studied how the representational resources involved in action simulation may be related to the resources involved in action execution and asked: does action execution affect the performance in occluded action tasks considered to reflect internal action simulation? In one of these studies, participants observed arm movements of a PLA while performing a corresponding arm movement themselves (Springer et al., 2011). The executed and observed movements were synchronized; furthermore, they were either fully congruent (i.e., involving the same anatomical body side and the same movement pattern; *full overlap*) or they were fully incongruent on both dimensions (*no overlap*), or they differed in either the anatomical body side used or in the movement pattern involved (*partial overlap*). For example, in a no overlap trial, the participant reached out his right arm to the right side, while the PLA lifted his left arm upwards over his head.

In each trial, the observed action was briefly occluded and then continued by the presentation of a static test pose (Graf et al., 2007). Participants indicated whether the test pose depicted a spatially coherent continuation of the previous arm movement. Two factors were manipulated: occluder time (the duration of occlusion) and pose time (the time at which the posture shown after occlusion was actually taken from the occluded movement), and each of them could take three values (100, 400 and 700 ms; as already explained in Section Simulation in Real Time: The Occluder Paradigm; Figure 1).

If real-time simulation takes place, response accuracy should be best when the occluder time (OT) and the pose time (PT) match, because then the internal representation (updated in real-time) should match the actual test pose (Graf et al., 2007). In addition, performance should yield a *monotonic distance function*, which emerges from the three levels of absolute time distances between the OT and PT (i.e., 0, 300 and 600 ms). If the two times match perfectly (i.e., no time distance), the test posture is presented just in time. Running a real-time simulation of the occluded action means an internal representation is run and updated, which can be used as a reference for evaluating the upcoming test pose. If real-time simulation occurs, that internal reference would, in the 0 ms distance condition, precisely match the test pose—whereas that match should be weaker in the conditions with a temporal distance of either 300 or 600 ms. This is reflected by a monotonic distance function, that is, a monotonic decrease of response accuracy with increasing temporal distance (e.g., Graf et al., 2007; Springer and Prinz, 2010). This description of the logic of the occluder paradigm by Graf et al. (2007) is a more technical recapitulation of the description already given earlier on in Section Simulation in Real Time: The Occluder Paradigm.

If internal simulation involves motor resources, the distance function should vary depending on the conditions of motor execution. This was, in fact, indicated. A monotonic distance effect (indicating real-time simulation) emerged when the observer's own movements were similar (but not identical) to the PLA's movements (i.e., partial overlap). In contrast, there was no monotonic distance effect for full overlap and no overlap (i.e., when both movements involved the same body sides and movement patterns and different body sides and movement patterns, respectively). This finding suggests that the degree of a representational overlap between performed and observed actions (e.g., Hommel et al., 2001) influenced the action simulation, as indicated by a monotonic distance effect.

However, spatial congruence may matter (Craighero et al., 2002; Kilner et al., 2009). That is, in one of the conditions of partial overlap, executed and observed movements involved the same movement pattern and occurred at the same side of the screen. This condition clearly showed a monotonic distance effect (i.e., real-time simulation). Hence, spatial congruence may have acted to increase the likelihood with which the participants engaged in internal action simulation when solving the task.

To test this alternative, an additional experiment was run in which participants were instructed that they would see the back view of the PLA, while all other parameters remained constant. This was possible because the PL stimuli being used were ambiguous with regard to front vs. back view. While under front view conditions, spatial and anatomical body side congruence falls apart, the back view manipulation implies that spatial and anatomical congruence corresponds, meaning that if the PLA and the executed action involve the same body side (e.g., left arm), they occur on the same side of the screen (left side). Hence, if spatial congruence matters, a monotonic distance function should occur in this condition.

However, the back view instructions revealed the same pattern as was found under front view instructions (Springer et al., 2011; Experiment 2). Specifically, the *mirror-inverted* constellation (implying spatial congruence between executed and observed movements) did not show a monotonic distance function. Therefore, the findings clearly contradicted a spatial congruence account. This study suggests that action simulation engages motor resources. The strength of the motor influences may depend on the amount of structural overlap between observed and executed actions (as defined by the anatomical side of the body and the movement pattern involved).

Further evidence of this view comes from a study by Tausche et al. (2010) examining effector-specific influences on the prediction of partly occluded full-body actions of a PLA (cf. Graf et al., 2007). While the movements observed were performed with either the arms or the legs, the participants themselves responded with a (different) movement involving either their arms or legs. The results indicated that a correspondence between the effectors observed and the effectors used induced a motor interference effect. Specifically, a monotonic distance effect, indicating real-time simulation, emerged for the incompatible trials (involving different effectors, i.e., arms and legs), whereas no such function occurred for the compatible trials (involving the same effectors, i.e., arms or legs).

Overall, these findings suggest that the accuracy with which an acting observer predicts others' actions may be influenced by anatomical mappings between performed and observed actions (Wapner and Cirillo, 1968; Sambrook, 1998; Gillmeister et al., 2008; Liepelt et al., 2010). This influence may arise at the level of effector-specific formats (Tausche et al., 2010; Springer et al., 2011; cf. Springer et al., 2013). This view accords with the notion that action observation activates the motor system in a corresponding somatotopic manner (Decety and Grèzes, 1999, 2006; Buccino et al., 2001; Grèzes and Decety, 2001).

### Dynamic and static processes

As the above-described studies demonstrated, physical activity does not appear to prevent participants from solving the action occlusion task (Tausche et al., 2010; Springer et al., 2011). Hence, additional and/or alternative processes may contribute to solving this task. Can action simulation recruit additional processes that are less motor-based when motor representations are constrained by execution?

It has, in fact, been suggested that predicting actions over visual occlusions may base on (at least) two different mental operations: dynamic updating and static matching (Springer and Prinz, 2010). Dynamic updating corresponds to an internal real-time simulation that should be indicated by a monotonic distance effect (i.e., performance should be best for time distances of 0 ms and should monotonically decrease for time distances of 300 and 600 ms; as explained previously; cf. Section Simulation in Real Time: The Occluder Paradigm). In the following, we use the term *real-time simulation* (specifying the timing of an assumed internal simulation process) synonymously with *dynamic simulation* and *dynamic updating*.

In addition to dynamic updating, performing an action occlusion task may involve a matching process, implying that the test pose after occlusion is matched against a statically maintained representation derived from the last action pose seen or perceived prior to occlusion (Springer and Prinz, 2010). If static matching takes place, performance in the action occlusion paradigm should decrease with increasing pose times (i.e., 100, 400, or 700 ms), irrespective of the actual duration of the occlusion period, because an increase in the pose time implies a decrease in the similarity between the last visible action pose (shown before occlusion) and the test pose (shown after occlusion) by definition. Hence, while static matching in its pure form predicts a main effect of pose time (but no interaction of occluder time and pose time and, therefore, no monotonic distance function), real-time simulation, in its pure form, predicts a strong interaction (emerging as a monotonic distance function), but no main effect of the pose time factor.

A study by Springer et al. (2013) used body part priming to address this issue. The participants played a motion-controlled video game for 5 min with either their arms or legs, yielding conditions of compatible and incompatible effector priming relative to subsequently performed arm movements of a PLA. The visual actions shown were briefly occluded after some time (action duration of 1254–1782 ms), followed by a static test pose. Participants judged whether or not the test pose showed a spatially coherent continuation of the previous action (as explained previously; cf. Graf et al., 2007). While compatible effector priming (e.g., arms) revealed evidence of dynamic updating (i.e., a monotonic distance effect, but no pose time effect), incompatible effector priming (e.g., legs) indicated static matching (i.e., a pose time effect, but no monotonic distance function). That is, in the compatible effector priming condition, response accuracy was best when the duration of occlusion matched the actual test pose shown after the occlusion, indicating an internal representation of the observed action was updated in real-time, thus matching the actual test pose. In addition, response accuracy decreased monotonically with increasing time difference between the duration of occlusion and the actual test pose (i.e., monotonic distance effect), corresponding to an increase of the time difference between an internal real-time model and the actual action outcome shown in the test pose. Hence, the findings of the compatible condition supported real-time simulation (Graf et al., 2007).

On the other hand, in the incompatible effector priming condition, evidence of real-time simulation was lacking (i.e., the duration of occlusion did not interact with the actual action progress shown in the test pose; see Figure 1). In this condition, however, response accuracy decreased with an increase in the pose time factor, implying a decrease in the similarity between the last visible action pose seen prior to occlusion and the test pose seen after occlusion—irrespective of the actual duration of the occlusion period. Thus, after being primed with incompatible effectors, participants were more accurate in the action occlusion task when the test pose shown was more similar to the most recently perceived action pose seen prior to occlusion (pose time effect). This effect cannot be explained by internal updating of the last perceived action image. It supports static matching. Instead of matching the test poses against real-time updated representations, participants in this condition may have alternatively matched the test poses against statically maintained representations derived from the most recently perceived action pose, which were maintained and then used as a static reference for the match with the upcoming test pose (Springer et al., 2013 cf. Springer and Prinz, 2010).

These results suggest that recognizing and predicting others' actions engages two distinct processes: dynamic updating (simulation) and static matching. The degree to which each process is involved may depend on contextual factors, such as the compatibility of the body parts involved in one's own and others' actions. Converging evidence comes from studies with a quite different focus of interest, for example, studies using semantic priming as a means of experimental context manipulation and addressing verbal descriptions of meaningful actions, rather than the kinematics involved in those actions.

### Semantic processes

The experiments we are going to consider now investigated the relationships between the processes involved in predicting occluded actions and those involved in semantic processing of verbal contents (Springer and Prinz, 2010; Springer et al., 2012; cf. Prinz et al., 2013). A great deal of previous research has indicated that motor processes are involved during the understanding of language that describes action (e.g., Pulvermüller, 2005, 2008; Andres et al., 2008; Fischer and Zwaan, 2008). For instance, while words denoting “far” and “near” printed on objects to be grasped yielded comparable effects on movement kinematics to the actual greater or shorter distances between hand position and the object (Gentilucci et al., 2000), processing verbal descriptions of actions activated compatible motor responses (e.g., Glenberg and Kaschak, 2002; Glenberg et al., 2008) and supported the conduct of reaching movements when the verb was processed prior to movement onset (Boulenger et al., 2006).

To what extent would verbal primes modulate the internal simulation of actions under conditions of temporary occlusion? In one study, the occluded action task was always preceded by a lexical decision task (Springer and Prinz, 2010; Experiment 2). Specifically, the participants judged whether a single word (onset 1250 ms) was a valid German verb (which was the case in 75% of trials, whereas pseudo-verbs appeared in the remaining 25 %). While all 102 verbs shown (all of them in the infinitive form) described achievable full-body actions, one half expressed high motor activity (like springen—“to jump”) while the other half expressed low motor activity (like *stehen*—“to stand”). This (relative) distinction of high vs. low motor activity resulted from an independent rating of the words by 20 volunteers.

On each trial, the lexical decision task was immediately followed by an occluded action task (as described previously) displaying a familiar PLA involving the whole body (e.g., lifting something from the floor, putting on a boot, or getting up from a chair). Instructions for the two tasks were given to make them appear to be completely unrelated to each other. However, as the results clearly showed, verbal content affected performance in this task. While lexical decisions involving high-activity verbs revealed a pronounced monotonic distance function (taken as a signature of internal real-time simulation), no such effect emerged for trials involving lexical decisions about low-activity verbs. We took these results as first evidence for the idea that the processes involved in an occluded action task may be tuned by the dynamic qualities of action verbs. To test this assumption, we ran another experiment in which the same verbs were used, but they were further differentiated according to the speed being expressed by “fast,” “moderate,” and “slow” action verbs based on an additional word rating (e.g., “to catch,” “to grasp,” “to stretch,” respectively; Springer and Prinz, 2010; Experiment 3). While words expressing fast and moderate actions produced a monotonic distance effect (indicating real-time simulation), slow action words clearly did not. That is, when the action occlusion task followed lexical decisions about verbs denoting fast and moderately fast actions, the monotonic distance function turned out to be more pronounced and steeper compared to trials in which the task was preceded by lexical decisions involving slow activity verbs.

These experiments suggest that language-based representations can affect the processes used for predicting actions observed in another individual. However, because the prime verbs always required lexical decisions, participants may have noticed that some of the verbs matched the visual actions, while others did not. Therefore, when responding to the test poses after occlusion (i.e., deciding whether or not it depicted a coherent continuation of the action), participants may have been more likely to give “yes” responses after a “match” than a “mismatch” (e.g., Forster and Davis, 1984).

To control for such strategy-based effects, we ran an additional experiment in which the prime verbs were masked and did not require any response at all (Springer et al., 2012). Specifically, ten verbs that had been rated as very fast (e.g., fangen—“to catch”) and ten verbs rated as very slow (e.g., lehnen—“to lean”) were briefly presented (onset 33 ms) embedded within a forward and a backward mask consisting of meaningless letter strings. Hence, people were not consciously aware of the verbal primes and were unlikely to engage in any deliberate response strategies (e.g., mapping the semantic content to the observed actions; see Forster, 1998; Van den Bussche et al., 2009). Still, masked priming revealed a similar result: While a pose time main effect was always present, indicating static matching was involved, a pronounced monotonic distance effect (taken to reflect dynamic updating, i.e., real-time simulation) emerged for verbs expressing dynamic actions, while it was lacking for verbs expressing static actions and meaningless letter strings (Springer et al., 2012).

While masked words are not visible, they have still been shown to access semantic processing levels (Kiefer and Spitzer, 2000; Schütz et al., 2007; Van den Bussche and Reynvoet, 2007). Also, when we used a non-semantic, purely visual priming of action dynamics (by presenting dots rotating with slow, moderate, or fast speed), a monotonic distance effect was lacking. Overall, the observations from both conscious and unconscious priming experiments seem to suggest that the semantic content implied in verbal processing has an impact on procedural operations involved in a subsequent occluded action task.

To better understand the nature of these effects the details of the putative internal action representation during occlusions and its underlying mental operations described above must be considered. Specifically, predicting occluded actions seems to imply two processes: dynamic updating and static matching. Hence, the observation that the slope of the monotonic distance function (indicating dynamic updating) is more pronounced after processing high-activity, as compared to low-activity action verbs, suggests (at least) two different functional interpretations (cf. Prinz et al., 2013). One is to consider a direct impact of verbal semantics on simulation dynamics—in the sense that the degree of activity expressed in the verbs affects the speed of simulation (faster after processing high-activity verbs as compared to low-activity verbs). The other alternative is that the distance function actually reflects a blend of performance resulting from two ways of solving the task: dynamic updating and static matching. While dynamic updating relies on real-time updating of the internal reference against which the test pose is matched, static matching relies on an internal reference that is static and may be derived from the last posture seen before occlusion (Springer and Prinz, 2010; Prinz et al., 2013).

Based on a direct differentiation of the two processes, as described previously (Springer et al., 2013), we argue that the results by Springer et al. (2012) can be best understood as a blend of outcomes of static and dynamic processes. That is, semantic verb content may modulate the relative contributions of two processes, static matching and dynamic updating. While high-activity verb contents invite stronger contributions of dynamic processing than low-activity contents, low-activity contents may promote stronger contributions of static processing.

In sum, the results from both explicit and implicit semantic priming experiments suggest that semantic verbal contents may impact on the mental operations involved when observers engage in recognizing actions that are transiently covered from sight (Springer and Prinz, 2010; Springer et al., 2012). Further studies converge with this view, although addressing semantic interference rather than priming effects (e.g., Liepelt et al., 2012; Diefenbach et al., 2013). For example, Liepelt et al. (2012) found evidence of interference between language and action, demonstrating that word perception influences hand actions and hand actions influence language production. Overall, one may conclude that internal action simulation and semantic processing can access common underlying representations, a view that corresponds to recent accounts of embodied cognition (e.g., Barsalou, 2003, 2008; Zwaan, 2004; Pulvermüller, 2005, 2008; Glenberg, 2008; Mahon and Caramazza, 2008, 2009).

## A framework for action representation

This paper focuses on experimental research investigating action simulation through systematic manipulation of the factors that influence how we perceive and predict actions observed in other people. While the studies discussed here differ according to a number of methodological aspects, including postdictive and predictive types of measurements, as well as the features studied, including the time course, sub-processes, and representational grounds of action simulation, all of them involve variations of an action occlusion paradigm (Graf et al., 2007; Prinz and Rapinett, 2008). This paradigm requires observers to evaluate the course of actions that are briefly and transiently covered from sight. When visual input is lacking, observers need to strongly rely on internally guided action representations. Thus, the paradigm allows for systematic testing of the cognitive underpinnings of action simulation and its internal processes and resources.

Several new insights emerged from the findings of these action occlusion paradigms. First, action simulation enables observers to render quite precise real-time predictions of others' actions (Graf et al., 2007; Parkinson et al., 2011; Sparenberg et al., 2012). For instance, observers were highly accurate in differentiating between time-coherent and time-incoherent continuations of temporarily occluded human full-body actions (Sparenberg et al., 2012) and spatially occluded human hand actions (Prinz and Rapinett, 2008). Hence, action simulation may involve an internal predictive process that runs in real-time with observed actions. This process may act on newly created action representations rather than relying on continuous visual extrapolations of observed movement trajectories (Prinz and Rapinett, 2008).

Second, action simulation seems to be highly susceptible to subtle visual manipulations, indicating that it draws on perceptual representations of diverse aspects of human motion and kinematic features, which may enable observers to develop highly accurate predictions about actions observed even after quite short phases of visual observation (Parkinson et al., 2012).

Third, action simulation can be influenced by an observer's own physical activity. Thus, the representational resources involved in internal action simulation may be related to the resources involved in motor execution. The strength of the motor influences varied according to the degree of correspondence between observed and performed actions, for instance, regarding the effectors involved (Tausche et al., 2010; Springer et al., 2011).

Fourth, predicting actions through periods of occlusion may involve two distinct processes: dynamic updating and static matching. While dynamic updating corresponds to real-time simulation, static matching implies that recently perceived action images are maintained as an internal reference against which newly incoming action information can be matched. The relative proportion to which the two processes are used may depend on contextual factors such as a correspondence of body parts involved in performed and perceived action (Springer et al., 2013).

Fifth, internal action simulation was affected by linguistic processing of action-related words. While prime verbs describing dynamic actions corresponding to the observed actions (i.e., implying movement of the limbs) revealed evidence of dynamic updating, this was not the case for those describing static actions (implying no movement of the limbs) (Springer and Prinz, 2010). This occurs even if people are not consciously aware of these action verbs and, thus, not prone to deliberate response strategies, suggesting that action simulation may involve semantic representational resources (Springer et al., 2012).

In the next section we are going to place the experimental evidence in the wider context of major theoretical issues in the broad domain of action and event representation.

### Real-time simulation and predictive coding

Several studies in which observers had to predict temporarily occluded actions have shown that prediction accuracy was best when the actions reappeared in a time-consistent manner after occlusions. In addition, prediction accuracy systematically decreased as the time gap between the duration of occlusion and the temporal advance of the action stage shown after occlusion increased (Graf et al., 2007; Springer and Prinz, 2010). These findings correspond to the notion that action simulation involves internal models that run in real-time with observed action (Verfaillie and Daems, 2002; Flanagan and Johansson, 2003; Rotman et al., 2006). Furthermore, internal real-time simulation was affected by the observers' own physical activity (Tausche et al., 2010; Springer et al., 2011).

Possible explanations for these results come from a predictive coding account of motor control (e.g., Kilner et al., 2007, 2009) and from the broader Theory of Event Coding (TEC; Prinz, 1990, 1997, 2006; Hommel et al., 2001). Efficient visuo-motor control requires estimating one's own body state prior to movement execution, which is based on internal forward models. These internal forward models allow individuals to anticipate the sensory consequences of their own movements in real-time based on motor commands (i.e., efference copies; Wolpert and Flanagan, 2001). They may also operate when observers engage in predicting actions observed in others (Grush, 2004; Blakemore and Frith, 2005; Prinz, 2006; Thornton and Knoblich, 2006; Kilner et al., 2007). Internal sensorimotor simulations may contribute to perceptual processing by generating top-down expectations and predictions of the unfolding action, allowing to precisely anticipating others' actions (see Wilson and Knoblich, 2005, for a review).

According to TEC, codes of perceived events and planned actions share a common representational domain. Perceptual codes and action codes may, thus, influence each other on the basis of this representational overlap. For instance, during different motor cognitive tasks (i.e., action observation or motor imagery), the cortical representations of a target muscle and a functionally related muscle were enhanced within a single task and across different tasks, suggesting a topographical and functional overlap of motor cortical representations (Marconi et al., 2007; cf. Dinstein et al., 2007). This overlap may provide a basis for anticipating others' actions by mapping those actions onto one's own sensorimotor experiences (Jeannerod, 2001; Gallese, 2005).

The participants in the experiments reported here (Section Representational Mechanisms) may have applied the same motor representations that were activated during execution to predicting a corresponding action observed. If so, the requirement to internally simulate an observed action may be reduced when observed and concurrently performed actions fully correspond, because under this condition execution by itself may already provide a continuously updated internal reference by which the occlusion task can be solved. Hence, this condition yielded better task performance than conditions in which observed and performed actions were not (or only partially) similar to each other (Springer et al., 2011). Given a complete lack of correspondence, execution may strongly interfere with internal simulation (Prinz, 1997; Wilson and Knoblich, 2005) such that internal simulations need to be shielded from information available from executing a movement that is entirely different from the observed one. Hence, this condition did not reveal evidence of internal simulation but showed increased errors, suggesting interference from execution to simulation (cf. Tausche et al., 2010). In fact, running real-time simulations of observed actions may be efficient for solving the task only when executed and observed movements are similar (but not when they are identical or fully incongruent on each possible dimension) (Springer et al., 2011). Here, evidence of real-time simulation was obtained, suggesting that the cost/benefit ratio for running internal sensorimotor simulations was more balanced, whether this is due to congruence in terms of the anatomical body sides used (Wilson and Knoblich, 2005) or the exact movement patterns involved in observed and performed actions (Kilner et al., 2003).

In line with this view, the temporal predictions generated by one's own motor system for efficient motor control may also be applied when predicting other people's actions (Blakemore and Frith, 2005; Kilner et al., 2007). Observers are able to quite precisely predict not only the sensory consequences of their own actions, but also those of others' actions (e.g., Sato, 2008). Furthermore, based on the observation of the communicative gestures of an agent in dyadic interaction, they are able to render quite precise predictions about when the action of the second agent will take place (Manera et al., 2013).

Neuroscientific studies have clearly shown the involvement of motor brain regions in action observation (e.g., Gallese et al., 1996; for a review see Iacoboni and Dapretto, 2006). This corresponds to the notion that an observer uses his or her motor system to simulate and predict others' actions (i.e., internal modeling on the basis of the observer's own sensorimotor experiences; e.g., Jeannerod, 2001; see Schubotz, 2007, for a review). When observers predicted transiently occluded full-body actions, different parts of the action observation network, including the dorsal premotor cortex, were involved (Stadler et al., 2011). Furthermore, grasp observation yielded increased activation of this network, including the dorsal premotor cortex and posterior parietal brain regions, which may reflect a motor simulation process for object-directed hand actions observed (Ramsey et al., 2012). Moreover, observing the start and middle phases of an action sequence yielded higher motor facilitation than observing the final postures of these actions (Urgesi et al., 2010), suggesting that parts of the human motor system are preferentially activated by predictive sensorimotor simulations of actions observed in other people (Blakemore and Frith, 2005; Kilner et al., 2007).

### Dynamic updating and static matching

Several experiments have indicated that two distinct processes may be involved when observers engage in predicting the future course of other people's actions: dynamic updating (corresponding to real-time simulation) and static matching (Section Representational Mechanisms). The relative contributions of dynamic and static processes may depend on contextual factors. For example, while priming the same effectors as perceived in another person revealed evidence of dynamic updating, priming incompatible effectors clearly did not (Springer et al., 2013). After incompatible effector priming, however, observers were better able to predict an occluded action when the action stage shown after occlusion was more similar to the most recently perceived action pose (seen prior to occlusion). This effect cannot be explained by internal real-time updating. It supports static matching. Instead of being matched against real-time updated internal models, test poses may, alternatively, be matched against statically maintained representations derived from the most accessible action pose, which are maintained and then used as a static reference for the match with the upcoming action.

Adopting a common coding perspective (TEC; Prinz, 1990, 1997; Hommel et al., 2001; Prinz and Hommel, 2002), participants may have mapped the (sensorimotor) representations used for acting to solve the action occlusion task. If action representations that were recently accessed could be mapped onto the actions perceived due to common representational grounds (i.e., due to effector compatibility), dynamic updating may be strengthened because recently activated internal real-time models (used for controlling one's own actions) can be mapped onto the perceived actions. Hence, using a compatible (but not incompatible) effector may aid action prediction (Reed and McGoldrick, 2007) and may foster internal real-time simulation (Springer et al., 2011).

On the other hand, if recently accessed action representations are not (or are less efficiently) applicable to internal forward models of perceived actions (due to effector incompatibility), real-time simulation may be constrained (Prinz, 1990, 1997; Hommel et al., 2001). Hence, incompatible effector priming fosters static matching as an alternative process for solving the action occlusion task, that is, matching internally stored action images without the involvement of (possibly conflicting) internal real-time models (Springer et al., 2013).

Corresponding to this view, observers were generally more accurate at predicting occluded actions after compatible than incompatible body part priming (Springer et al., 2013). This finding may suggest that real-time simulations yielded, overall, more precise predictions than static matching. This view corresponds to the notion that internal sensorimotor activation (simulations) are used when predicting others' actions (Blakemore and Frith, 2005; Wilson and Knoblich, 2005; Kilner et al., 2009) and that action observation activates premotor brain regions in a somatotopic way (i.e., reflecting the body parts being observed; Decety and Grèzes, 1999, 2006; Buccino et al., 2001; Sakreida et al., 2005).

### Action semantics

Several experiments indicated that the precision by which observers were able to predict the future course of an action was affected by verbal primes (Section Semantic Processes). One intriguing explanation for this is to assume that language-based descriptions of actions may modulate the relative involvement of two processes: dynamic updating (i.e., real-time simulations) and static matching (as explained previously).

A large body of evidence shows that processing verbal information is closely linked to information processing in sensory and motor domains, indicating that activation of semantic knowledge coincides with activation of corresponding sensory and/or motor representations (Barsalou, 2003, 2008; Barsalou et al., 2003; Glenberg, 2008; Kiefer et al., 2008; Pulvermüller, 2005, 2008; Mahon and Caramazza, 2008, 2009). Likewise, many studies have indicated that motor control may be closely linked to semantic processing, such that the kinematics of ongoing movements are affected by semantic processing (Gentilucci et al., 2000; Glover et al., 2004; Boulenger et al., 2006, 2008).

Related to the studies reported here (Section Semantic Processes), one may assume that verbs describing dynamic action and implying movement of the limbs (corresponding to the observed actions) act to strengthen the involvement of dynamic updating over static matching due to common representational grounds between meaning and movement (Barsalou, 2003, 2008; Pulvermüller, 2005; Glenberg, 2008). As a result, dynamic updating was indicated when participants accessed verbs expressing a dynamic action prior to an action occlusion task. Correspondingly, static action verbs, which did not imply movement of the limbs, did not indicate dynamic updating. Static (and meaningless) primes may have favored the contribution of static matching, thus, preventing an indication of dynamic updating from occurring (Springer and Prinz, 2010; Springer et al., 2012).

This pattern was even observed when people were not aware of the primes and were, thus, unlikely to have engaged in deliberate task strategies (Springer et al., 2012). When the verbal descriptions involved a coding of action dynamics that corresponded to the visual actions, dynamic real-time simulation was indicated. Hence, linguistic representations may trigger anticipatory internal simulations, thus affecting the processes involved in an action prediction task (Springer and Prinz, 2010; Springer et al., 2012).

Overall, the observation of a semantic modulation of action simulation converges with recent evidence supporting the notion of close links between semantic processing and internal action simulation (Liepelt et al., 2012; Diefenbach et al., 2013). This view is consistent with embodied accounts, which hold that understanding action language coincides (or even requires) internal sensorimotor simulations (or reactivation) of the described action. In these theories, sensorimotor simulation is understood as the activation of the same representations (and neural structures) that are derived from bodily experience, but in the absence of overt performance (e.g., Glenberg and Kaschak, 2002; Barsalou et al., 2003; Zwaan, 2004; Pulvermüller, 2005; Zwaan and Taylor, 2006; Barsalou, 2008; see Rumiati et al., 2010, for a review).

Recent evidence has clearly demonstrated cross-talk effects between action language and execution (e.g., Nazir et al., 2008). Processing action verbs modulated the kinematics of movements relative to nouns without motor associations (Boulenger et al., 2006). Parts of the motor system were activated when words and sentences implying the corresponding actions (e.g., the same effector) were perceived (Buccino et al., 2001; Aziz-Zadeh et al., 2006). Pulvermüller et al. (2005) found somatotopic activity in the motor cortex when participants were listening to face- and leg-related action words; corresponding to the view that motor regions of the brain are involved in action word retrieval (Pulvermüller, 2005). Furthermore, reading hand-related action verbs conjugated in the future enhanced the excitability of hand muscles relative to reading the same verbs conjugated in the past tense; indicating that an activation of predictive sensorimotor simulations is not restricted to direct action observation but may also be induced by action-related features derived from linguistic stimuli (Candidi et al., 2010).

### Limitations, open questions, future directions

One conclusion from several studies discussed in this paper is that one mechanism by which a given action perception context can modulate the precision of internal predictions about the future course of other people's actions is by altering the relative contributions of dynamic and static processes. While dynamic updating corresponds to an internal predictive simulation process, static matching implies that most recently accessed action representations are maintained and then retrospectively used for evaluating newly incoming information (e.g., Springer and Prinz, 2010; Springer et al., 2013). However, although this model seems to fit several of the studies we have discussed here, there are some limitations and open issues to consider.

Firstly, neither of the two processes by themselves speaks to the representational modality to which the operations pertain (e.g., updating/matching in the visual and/or motor domain). Possibly, predicting occluded actions may not rely on only one representational domain but may involve alternating or simultaneous processes in different domains (e.g., visual driven static matching and motor driven dynamic simulation). Likewise, the order in which the two processes may run (e.g., trial-by-trial or in parallel) is, at this point, an open question that needs to be addressed in future work.

Secondly, the accuracy of predicting (simulating) actions may be moderated by individual characteristics such as age or sensorimotor expertise. While many studies have shown that higher motor expertise goes along with stronger motor simulation during observation of actions from the respective domain of expertise (Calvo-Merino et al., 2005; Cross et al., 2006; Aglioti et al., 2008; Urgesi et al., 2012), only few studies have illuminated how the aging process might interact with sensorimotor expertise during action prediction (Diersch et al., 2012, 2013). Diersch et al. (2012) found that figure skating expertise can improve both young and older adults' action prediction abilities when those actions are within the observer's domain of physical expertise. Thus, sensorimotor expertise, even when acquired many decades ago, may still strongly impact our ability to precisely predict others' actions. Thirdly, the interpersonal relationship between an observer and the agent observed may matter. This may concern close relationships (e.g., children, parents, or romantic partners) and novel social partners (e.g., strangers). Taking self-generated actions as an extreme illustration of actions to which observers have privileged access, it has been shown that observers are most accurate in predicting those actions that they are able to perform themselves (e.g., Knoblich et al., 2002). Apart from allowing one to regulate one's own behavior, such privileged self-recognition enables recognition of the effects of one's own actions as being self-generated (Jeannerod, 1999, 2003; Frith et al., 2000).

Although the focus scope of the current paper was quite narrow, in that it focused on action simulation, experimentally investigated by behavioral action occlusion paradigms, considering other strands of action simulation research, like modelling studies (e.g., Fleischer et al., 2012) or studies focusing on the processing of robot vs. humanoid form and motion (e.g., Saygin and Stadler, 2012; see Gowen and Poliakoff, 2012, for a review), may complement the work discussed here.

On a neuroscientific level, investigating the involvement of common and/or distinct brain networks in relation to the different processes engaged in action prediction seems to be highly promising (e.g., Schiffer and Schubotz, 2011; Ramsey et al., 2012; cf. Szpunar et al., 2013). Yet, only few human fMRI studies have examined action simulation by use of action occlusion paradigms (Stadler et al., 2011; Diersch et al., 2013). In line with our notion that predicting others' actions recruits dynamic and static processes, Stadler et al. (2011) found that different portions of the premotor cortex play different roles in each of these aspects. While the right pre-supplementary motor area (pre-SMA) was recruited for maintaining an internal reference of transiently occluded actions, dynamic updating of internal action representations yielded increased activation in the pre-SMA and the dorsal premotor cortex (PMd) (Stadler et al., 2011; see also Stadler et al., 2012a).

In sum, the studies we have discussed in this paper collectively suggest that action simulation can be conceived of as a highly susceptible, dynamic process that runs in real-time with actions observed, involving sensorimotor and semantic representations. Moreover, when predicting the future course of other people's actions, dynamic simulations may co-exist with similarity-based evaluations of statically maintained action representations (static matching). The relative involvement of both processes, dynamic simulation and static matching, may be tuned by contextual factors, like understanding action-related verbal contents, or actually performing actions corresponding to those observed in other people. This view corresponds to the general assumption that an observer can use his or her own motor system to internally simulate and predict others' actions (Grèzes and Decety, 2001; Jeannerod, 2001) and is compatible with a more specific predictive coding account of motor control (e.g., Kilner et al., 2007).

### Conflict of interest statement

The authors declare that the research was conducted in the absence of any commercial or financial relationships that could be construed as a potential conflict of interest.

## References

[B1] Aglioti S. M. Cesari P. Romani M. Urgesi C. (2008). Action anticipation and motor resonance in elite basketball players. Nat. Neurosci. 11, 1109–1116 10.1038/nn.2182 19160510

[B2] Andres M. Olivier E. Badets A. (2008). Action, words and numbers: a motor contribution to semantic processing? Curr. Dir. Psychol. Sci. 17, 313–317 10.1111/j.1467-8721.2008.00597.x

[B3] Aziz-Zadeh L. Wilson S. M. Rizzolatti G. Iacoboni M. (2006). Embodied semantics and the premotor cortex: congruent representations for visually presented actions and linguistic phrases describing actions. Curr. Biol. 16, 1818–1823 10.1016/j.cub.2006.07.060 16979559

[B4] Barsalou L. W. (2003). Abstraction in perceptual symbol systems. Philos. Trans. R. Soc. Lond. B Biol. Sci. 358, 1177–1187 10.1098/rstb.2003.1319 12903648 PMC1693222

[B5] Barsalou L. W. (2008). Grounded cognition. Annu. Rev. Psychol. 59, 617–645 10.1146/annurev.psych.59.103006.093639 17705682

[B6] Barsalou L. W. Simmons W. K. Barbey A. K. Wilson C. D. (2003). Grounding conceptual knowledge in modality-specific systems. Trends Cogn. Sci. 7, 84–91 10.1016/S1364-6613(02)00029-3 12584027

[B7] Blakemore S. J. Decety J. (2001). From the perception of action to the understanding of intention. Nat. Rev. Neurosci. 2, 561–567 10.1038/35086023 11483999

[B8] Blakemore S. J. Frith C. (2005). The role of motor contagion in the prediction of action. Neuropsychologia 43, 260–267 10.1016/j.neuropsychologia.2004.11.012 15707910

[B9] Boulenger V. Roy A. C. Paulignan Y. Deprez V. Jeannerod M. Nazir T. A. (2006). Cross-talk between language processes and overt motor behavior in the first 200 ms of processing. J. Cogn. Neurosci. 18, 1606–1615 10.1162/jocn.2006.18.10.1607 17014366

[B10] Boulenger V. Silber B. Y. Roy A. C. Paulignan Y. Jeannerod M. Nazir T. A. (2008). Subliminal display of action words interferes with motor planning: a combined EEG and kinematic study. J. Physiol. Paris 102, 130–136 10.1016/j.jphysparis.2008.03.015 18485678

[B11] Brass M. Bekkering H. Prinz W. (2001). Movement observation affects movement execution in a simple response task. Acta Psychol. 106, 3–22 10.1016/S0001-6918(00)00024-X 11256338

[B12] Buccino G. Binkofski F. Fink G. R. Fadiga L. Fogassi L. Gallese V. (2001). Action observation activates premotor and parietal areas in a somatotopic manner: an fMRI study. Eur. J. Neurosci. 13, 400–404 10.1046/j.1460-9568.2001.01385.x 11168545

[B13] Buccino G. Vogt S. Ritzi A. Fink G. R. Zilles K. Freund H.-J. (2004). Neural circuits underlying imitation learning of hand actions: an event-related fMRI study. Neuron 42, 323–334 10.1016/S0896-6273(04)00181-3 15091346

[B14] Calvo-Merino B. Glaser D. E. Grezes J. Passingham R. E. Haggard P. (2005). Action observation and acquired motor skills: an fMRI study with expert dancers. Cereb. Cortex 15, 1243–1249 10.1093/cercor/bhi007 15616133

[B15] Candidi M. Leone-Fernandez B. Barber H. A. Carreiras M. Aglioti S. M. (2010). Hands on the future: facilitation of cortico-spinal hand-representation when reading the future tense of hand-related action verbs. Eur. J. Neurosci. 32, 677–683 10.1111/j.1460-9568.2010.07305.x 20626461

[B16] Chartrand T. L. Bargh J. A. (1999). The chameleon effect: the perception-behavior link and social interaction. J. Pers. Soc. Psychol. 76, 893–910 10.1037/0022-3514.76.6.893 10402679

[B17] Craighero L. Bello A. Fadiga L. Rizzolatti G. (2002). Hand action preparation influences the responses to hand pictures. Neuropsychologia 40, 492–502 10.1016/S0028-3932(01)00134-8 11749979

[B18] Cross E. S. Hamilton A. F. D. C. Grafton S. T. (2006). Building a motor simulation de novo: observation of dance by dancers. Neuroimage 31, 1257–1267 10.1016/j.neuroimage.2006.01.033 16530429 PMC1821082

[B19] Cutting J. E. Moore C. Morrison R. (1988). Masking the motions of human gait. Percept. Psychophys. 44, 339–347 10.3758/BF03210415 3226881

[B20] Decety J. Grèzes J. (1999). Neural mechanisms subserving the perception of human actions. Trends Cogn. Sci. 3, 172–178 10.1016/S1364-6613(99)01312-1 10322473

[B21] Decety J. Grèzes J. (2006). The power of simulation: imagining one's own and other's behavior. Brain Res. 1079, 4–14 10.1016/j.brainres.2005.12.115 16460715

[B22] Diefenbach C. Rieger M. Massen C. Prinz W. (2013). Action-sentence compatibility: the role of action effects and timing. Front. Psychol. 4:272 10.3389/fpsyg.2013.00272 23734134 PMC3659315

[B23] Diersch N. Cross E. S. Stadler W. Schütz-Bosbach S. Rieger M. (2012). Representing others' actions: the role of expertise in the aging mind. Psychol. Res. 76, 525–541 10.1007/s00426-011-0404-x 22198511

[B24] Diersch N. Mueller K. Cross E. S. Stadler W. Rieger M. Schütz-Bosbach S. (2013). Action prediction in younger versus older adults: neural correlates of motor familiarity. PLoS ONE 8:e64195 10.1371/journal.pone.0064195 23704980 PMC3660406

[B25] Dinstein I. Hasson U. Rubin N. Heeger D. J. (2007). Brain areas selective for both observed and executed movements. J. Neurophysiol. 98, 1415–1427 10.1152/jn.00238.2007 17596409 PMC2538553

[B26] Doerrfeld A. Sebanz N. Shiffrar M. (2012). Expecting to lift a box together makes the load look lighter. Psychol. Res. 76, 467–475 10.1007/s00426-011-0398-4 22159762 PMC3383959

[B27] Enticott P. G. Johnston P. J. Herring S. E. Hoy K. E. Fitzgerald P. B. (2008). Mirror neuron activation is associated with facial emotion processing. Neuropsychologia 46, 2851–2854 10.1016/j.neuropsychologia.2008.04.022 18554670

[B28] Fischer M. H. Zwaan R. A. (2008). Embodied language: a review of the role of the motor system in language comprehension. Q. J. Exp. Psychol. 61, 825–850 10.1080/17470210701623605 18470815

[B29] Flanagan J. R. Johansson R. S. (2003). Action plans used in action observation. Nature 424, 769–771 10.1038/nature01861 12917683

[B30] Fleischer F. Christensen A. Caggiano V. Thier P. Giese M. A. (2012). Neural theory for the perception of causal actions. Psychol. Res. 76, 476–493 10.1007/s00426-012-0437-9 22535418

[B31] Forster K. I. (1998). The pros and cons of masked priming. J. Psychol. Res. 27, 203–233 10.1023/A:1023202116609 9561785

[B32] Forster K. I. Davis C. (1984). Repetition priming and frequency attenuation in lexical access. J. Exp. Psychol. Learn. Mem. Cogn. 10, 680–698 10.1037/0278-7393.10.4.680

[B33] Frith C. D. Blakemore S.-J. Wolpert D. M. (2000). Abnormalities in the awareness and control of action. Philos. Trans. R. Soc. Lond. B 355, 1771–1788 10.1098/rstb.2000.0734 11205340 PMC1692910

[B34] Gallese V. (2005). Embodied simulation: from neurons to phenomenal experience. Phenomenol. Cogn. Sci. 4, 23–48 10.1007/s11097-005-4737-z

[B35] Gallese V. Fadiga L. Fogassi L. Rizzolatti G. (1996). Action recognition in the premotor cortex. Brain 119, 593–609 10.1093/brain/119.2.593 8800951

[B36] Gallese V. Keysers C. Rizzolatti G. (2004). A unifying view of the basis of social cognition. Trends Cogn. Sci. 8, 396–403 10.1016/j.tics.2004.07.002 15350240

[B37] Gentilucci M. Benuzzi F. Bertolani L. Daprati E. Gangitano M. (2000). Language and motor control. Exp. Brain Res. 133, 468–490 10.1007/s002210000431 10985682

[B38] Gillmeister H. Catmur C. Liepelt R. Brass M. Heyes C. (2008). Experience-based priming of body parts: a study of action imitation. Brain Res. 1217, 157–170 10.1016/j.brainres.2007.12.076 18502404

[B39] Glenberg A. M. (2008). Toward the integration of bodily states, language, and action, in Embodied Grounding: Social, Cognitive, Affective and Neuroscientific Approaches, eds Semin G. R. Smith E. R. (New York, NY: Cambridge University Press), 43–70 10.1017/CBO9780511805837.003

[B40] Glenberg A. M. Kaschak M. P. (2002). Grounding language in action. Psychon. Bull. Rev. 9, 558–565 10.3758/BF03196313 12412897

[B41] Glenberg A. M. Sato M. Cattaneo L. Riggio L. Palumbo D. Buccino G. (2008). Processing abstract language modulates motor system activity. Q. J. Exp. Psychol. 1, 1–15 10.1080/17470210701625550 18470821

[B42] Glover S. Rosenbaum D. A. Graham J. Dixon P. (2004). Grasping the meaning of words. Exp. Brain Res. 154, 103–108 10.1007/s00221-003-1659-2 14578997

[B43] Goldman A. I. (2005). Imitation, mind reading, and simulation, in Imitation, Human Development, and Culture, eds Hurley S. Chater N. (Cambridge, MA: MIT Press), 79–93

[B44] Goldman A. I. (2006). Simulating Minds: The Philosophy, Psychology, and Neuroscience of Mindreading. New York, NY: Oxford University Press 10.1093/0195138929.001.0001

[B45] Gordon R. M. (1996). Radical' simulationism, in Theories of Theories of Mind, eds Carruthers P. Smith P. K. (Cambridge, UK: Cambridge University Press), 11–21 10.1017/CBO9780511597985.003

[B46] Gordon R. M. (2001). Simulation and reason explanation: the radical view. Philos. Top. 29, 175–192 10.5840/philtopics2001291/210

[B47] Gowen E. Poliakoff E. (2012). How does visuomotor priming differ for biological and non-biological stimuli? A review of the evidence. Psychol. Res. 76, 407–420 10.1007/s00426-011-0389-5 22302411

[B48] Graf M. Reitzner B. Corves C. Casile A. Giese M. Prinz W. (2007). Predicting point-light actions in real-time. Neuroimage 36, T22–T32 10.1016/j.neuroimage.2007.03.017 17499167

[B49] Grèzes J. Decety J. (2001). Functional anatomy of execution, mental simulation, observation, and verb generation of actions: a meta-analysis. Hum. Brain Mapp. 12, 1–19 10.1002/1097-0193(200101)12:1<1::AID-HBM10>3.0.CO;2-V 11198101 PMC6872039

[B50] Grush R. (2004). The emulation theory of representation: motor control, imagery, and perception. Behav. Brain Sci. 27, 377–442 10.1017/S0140525X04000093 15736871

[B51] Hamilton A. Wolpert D. Frith U. (2004). Your own action influences how you perceive another person's action. Curr. Biol. 14, 493–498 10.1016/j.cub.2004.03.007 15043814

[B52] Hommel B. Müsseler J. Aschersleben G. Prinz W. (2001). The theory of event coding (TEC). A framework for perception and action planning. Behav. Brain Sci. 24, 849–937 10.1017/S0140525X01000103 12239891

[B53] Iacoboni M. Dapretto M. (2006). The mirror neuron system and the consequences of its dysfunction. Nat. Rev. Neurosci. 7, 942–951 10.1038/nrn2024 17115076

[B54] Jeannerod M. (1999). The 25th Bartlett Lecture: to act or not to act: perspectives on the representation of actions. Q. J. Exp. Psychol. 52A, 1–29 10.1080/713755803 10101973

[B55] Jeannerod M. (2001). Neural simulation of action: a unifying mechanism for motor cognition. Neuroimage 14, 103–109 10.1006/nimg.2001.0832 11373140

[B56] Jeannerod M. (2003). The mechanism of self-recognition in humans. Behav. Brain Res. 142, 1–15 10.1016/S0166-4328(02)00384-4 12798261

[B57] Jellema T. Perrett D. I. (2003). Cells in monkey STS responsive to articulated body motions and consequent static posture: a case of implied motion? Neuropsychologia 41, 1728–1737 10.1016/S0028-3932(03)00175-1 14527537

[B58] Johansson G. (1973). Visual perception of biological motion and a model for its analysis. Percept. Psychophys. 14, 201–211 10.3758/BF03212378

[B59] Johansson G. (1976). Spatio-temporal differentiation and integration in visual motion perception. An experimental and theoretical analysis of calculus-like functions in visual data processing. Psychol. Res. 38, 379–93 10.1007/BF00309043 1005623

[B60] Kiefer M. Sim E.-J. Herrnberger B. Grothe J. Hoenig K. (2008). The sound of concepts: four markers for a link between auditory and conceptual brain systems. J. Neurosci. 28, 12224–12230 10.1523/JNEUROSCI.3579-08.2008 19020016 PMC6671691

[B61] Kiefer M. Spitzer M. (2000). Time course of conscious and unconscious semantic brain activation. Neuroreport 11, 2401–2407 10.1097/00001756-200008030-00013 10943693

[B62] Kilner J. M. Friston K. J. Frith C. D. (2007). Predictive coding: an account of the mirror neuron system. Cogn. Proc. 8, 159–166 10.1007/s10339-007-0170-2 17429704 PMC2649419

[B63] Kilner J. M. Marchant J. L. Frith C. D. (2009). Relationship between activity in human primary motor cortex during action observation and the mirror neuron system. PLoS ONE 4:e4925 10.1371/journal.pone.0004925 19290052 PMC2654140

[B64] Kilner J. M. Paulignan Y. Blakemore S.-J. (2003). An interference effect of observed biological movement on action. Curr. Biol. 13, 522–525 10.1016/S0960-9822(03)00165-9 12646137

[B65] Knoblich G. Seigerschmidt E. Flach R. Prinz W. (2002). Authorship effects in the prediction of handwriting strokes: evidence for action simulation during action perception. Q. J. Exp. Psychol. 55, 1027–1046 10.1080/02724980143000631 12188508

[B66] Liepelt R. Dolk T. Prinz W. (2012). Bidirectional semantic interference between action and speech. Psychol. Res. 76, 446–455 10.1007/s00426-011-0390-z 22075764

[B67] Liepelt R. Prinz W. Brass M. (2010). When do we simulate non-human agents? Dissociating communicative and non-communicative actions. Cognition 115, 426–434 10.1016/j.cognition.2010.03.003 20356576

[B68] Mahon B. Z. Caramazza A. (2008). A critical look at the embodied cognition hypothesis and a new proposal for grounding conceptual content. J. Physiol. 102, 59–70 10.1016/j.jphysparis.2008.03.004 18448316

[B69] Mahon B. Z. Caramazza A. (2009). Concepts and categories: a cognitive neuropsychological perspective. Annu. Rev. Psychol. 60, 27–51 10.1146/annurev.psych.60.110707.163532 18767921 PMC2908258

[B70] Manera V. Schouten B. Verfaillie K. Becchio C. (2013). Time will show: real time predictions during interpersonal action perception. PLoS ONE 8:e54949 10.1371/journal.pone.0054949 23349992 PMC3551817

[B71] Marconi B. Pecchioli C. Koch G. Caltagirone C. (2007). Functional overlap between hand and forearm motor cortical representations during motor cognitive tasks. Clin. Neurophysiol. 118, 1767–1775 10.1016/j.clinph.2007.04.028 17576095

[B72] Mukamel R. Ekstrom A. D. Kaplan J. Iacoboni M. Fried I. (2010). Single-neuron responses in humans during execution and observation of actions. Curr. Biol. 20, 750–756 10.1016/j.cub.2010.02.045 20381353 PMC2904852

[B73] Nazir T. A. Boulenger V. Roy A. Silber B. Jeannerod M. Paulignan Y. (2008). Language-induced motor perturbations during the execution of a reaching movement. Q. J. Exp. Psychol. 61, 933–943 10.1080/17470210701625667 18470823

[B74] Parkinson J. Springer A. Prinz W. (2011). Can you see me in the snow? Action simulation aids the detection of visually degraded human motion. Q. J. Exp. Psychol. 64, 1463–1472 10.1080/17470218.2011.594895 21812593

[B75] Parkinson J. Springer A. Prinz W. (2012). Before, during and after you disappear: aspects of timing and dynamic updating of the real-time action simulation of human motions. Psychol. Res. 76, 421–433 10.1007/s00426-012-0422-3 22349885

[B76] Prinz W. (1990). A common coding approach to perception and action, in Relationships between Perception and Action: Current Approaches, eds Neumann O. Prinz W. (Berlin: Springer), 167–201 10.1007/978-3-642-75348-0_7

[B77] Prinz W. (1997). Perception and action planning. Eur. J. Cogn. Psychol. 9, 129–154 10.1080/713752551

[B78] Prinz W. (2006). What re-enactment earns us. Cortex 42, 515–517 10.1016/S0010-9452(08)70389-7 16881261

[B79] Prinz W. Hommel B. (2002). Common Mechanisms in Perception and Action: Attention and Performance XIX. Oxford: Oxford University Press

[B80] Prinz W. Rapinett G. (2008). Filling the gap: dynamic representation of occluded action, in Enacting Intersubjectivity: A Cognitive and Social Perspective on the Study of Interactions, eds Morganti F. Carassa A. Riva G. (Amsterdam: IOS Press), 223–236 ISBN: 978-1-58603-850-2

[B81] Prinz W. Diefenbach C. Springer A. (2013). The meaning of actions. Cross-talk between procedural and declarative action knowledge, in Joint Attention and Agency, eds Terrace H. Metcalfe J. (New York, NY: Oxford University Press). (in press).

[B82] Pulvermüller F. (2005). Brain mechanisms linking language and action. Nat. Rev. Neurosci. 6, 576–582 10.1038/nrn1706 15959465

[B83] Pulvermüller F. (2008). Grounding language in the brain, in Symbols and embodiment. Debates on meaning and cognition, eds Vega M. D. Glenberg A. M. Graesser A. C. (New York, NY: Oxford University Press), 85–116 10.1093/acprof:oso/9780199217274.003.0006

[B84] Pulvermüller F. Shtyrov Y. Ilmoniemi R. (2005). Brain signatures of meaning access in action word recognition. J. Cogn. Neurosci. 17, 1–9 10.1162/0898929054021111 15969907

[B85] Ramsey R. Cross E. S. Hamilton A. F. de C. (2012). Predicting others' actions via grasp and gaze: evidence for distinct brain networks. Psychol. Res. 76, 494–502 10.1007/s00426-011-0393-9 22120203

[B86] Reed C. L. McGoldrick J. E. (2007). Action during body perception: processing time affects self-other correspondences. Soc. Neurosci. 2, 134–149 10.1080/17470910701376811 18633812

[B87] Rizzolatti G. Fadiga L. Gallese V. Fogassi L. (1996). Premotor cortex and the recognition of motor actions. Cogn. Brain Res. 3, 131–141 10.1016/0926-6410(95)00038-0 8713554

[B88] Rizzolatti G. Fogassi L. Gallese V. (2006). Mirrors in the Mind. Sci. Am. 295, 30–37 10.1038/scientificamerican1106-54 17076084

[B89] Rotman G. Troje N. F. Johansson R. S. Flanagan J. R. (2006). Eye movements when observing predictable and unpredictable actions. J. Neurophysiol. 96, 1358–1369 10.1152/jn.00227.2006 16687620

[B90] Ruby P. Decety J. (2001). Effect of subjective perspective taking during simulation of action: a PET investigation of agency. Nat. Neurosci. 4, 546–550 10.1038/87510 11319565

[B91] Rumiati R. I. Papeo L. Corradi-Dell'Acqua C. (2010). Higher-level motor processes. Ann. N.Y. Acad. Sci. 1191, 219–241 10.1111/j.1749-6632.2010.05442.x 20392283

[B92] Sakreida K. Schubotz R. I. Wolfensteller U. von Cramon D. Y. (2005). Motion class dependency in observer's motor areas revealed by functional magnetic resonance imaging. J. Neurosci. 25, 1335–1342 10.1523/JNEUROSCI.4170-04.2005 15703387 PMC6725994

[B93] Sambrook T. D. (1998). Does visual perspective matter in imitation? Perception 27, 1461–1473 10.1068/p271461 10505190

[B94] Sato A. (2008). Action observation modulates auditory perception of the consequences of others' actions. Conscious. Cogn. 17, 1219–1227 10.1016/j.concog.2008.01.003 18299207

[B95] Saygin A. P. Stadler W. (2012). The role of appearance and motion in action prediction. Psychol. Res. 76, 388–394 10.1007/s00426-012-0426-z 22371203

[B95a] Schiffer A.-M. Schubotz R. I. (2011). Caudate nucleus signals for breaches of expectation in a movement observation paradigm. Front. Hum. Neurosci. 5:38 10.3389/fnhum.2011.00038 21519392 PMC3078751

[B96] Schubotz R. I. (2007). Prediction of external events with our motor system: towards a new framework. Trends. Cogn. Sci. (Regul. Ed.) 11, 211–218 10.1016/j.tics.2007.02.006 17383218

[B97] Schütz K. Schendzielarza I. Zwitserlood P. Vorberg D. (2007). Nice wor_ if you can get the wor_: subliminal semantic and form priming in fragment completion. Conscious. Cogn. 16, 520–532 10.1016/j.concog.2006.09.001 17045810

[B98] Schütz-Bosbach S. Prinz W. (2007). Perceptual resonance: action-induced modulation of perception. Trends Cogn. Sci. 11, 349–355 10.1016/j.tics.2007.06.005 17629544

[B99] Sebanz N. Knoblich G. (2009). Prediction in joint action: what, when, and where. Top. Cogn. Sci. 1, 353–367 10.1111/j.1756-8765.2009.01024.x 25164938

[B100] Sparenberg P. Springer A. Prinz W. (2012). Predicting others' actions: evidence for a constant time delay in action simulation. Psychol. Res. 76, 41–49 10.1007/s00426-011-0321-z 21365343

[B101] Springer A. Prinz W. (2010). Action semantics modulate action prediction. Q. J. Exp. Psychol. 63, 2141–2158 10.1080/17470211003721659 20526978

[B102] Springer A. Brandstädter S. Prinz W. (2013). Dynamic simulation and static matching for action prediction: evidence from body part priming. Cogn. Sci. 10.1111/cogs.12044 23692214

[B103] Springer A. Brandstädter S. Liepelt R. Birngruber T. Giese M. Mechsner F. Prinz W. (2011). Motor execution affects action prediction. Brain Cogn. 76, 26–36 10.1016/j.bandc.2011.03.007 21477908

[B104] Springer A. Huttenlocher A. Prinz W. (2012). Language-induced modulation during the prediction of others' actions. Psychol. Res. 76, 456–466 10.1007/s00426-012-0411-6 22234446

[B107] Stadler W. Schubotz R. von Cramon D. Y. Springer A. Graf M. Prinz W. (2011). Predicting and memorizing observed action: differential premotor cortex involvement. Hum. Brain Mapp. 32, 677–687 10.1002/hbm.20949 20225220 PMC6870275

[B105] Stadler W. Ott D. V. M. Springer A. Schubotz R. I. Schütz-Bosbach S. Prinz W. (2012a). Repetitive TMS suggests a role of the human dorsal premotor cortex in action prediction. Front. Hum. Neurosci. 6:20 10.3389/fnhum.2012.00020 22363279 PMC3282473

[B106] Stadler W. Springer A. Parkinson J. Prinz W. (2012b). Movement kinematics affect action prediction: comparing human to non-human point-light actions. Psychol. Res. 76, 395–406 10.1007/s00426-012-0431-2 22411563

[B108] Szpunar K. K. St. Jacques P. L. Robbins C. A. Wig G. S. Schacter D. L. (2013). Repetition-related reductions in neural activity reveal component processes of mental simulation. Soc. Cogn. Affect. Neurosci. 10.1093/scan/nst035 23482621 PMC4014108

[B109] Tausche P. Springer A. Prinz W. (2010). Effector-specific motor interference in action simulation, in Proceedings of the 32nd Annual Conference of the Cognitive Science Society, eds Ohlsson S. Catrambone R. (Austin, TX: Cognitive Science Society), 2698–2703

[B110] Thirkettle M. Benton C. P. Scott-Samuel N. E. (2009). Contributions of form, motion and task to biological motion perception. J. Vis. 9, 28.1–28.11 10.1167/9.3.28 19757967

[B111] Thornton I. M. Knoblich G. (2006). Action perception: seeing the world through a moving body. Curr. Biol. 16, R27–R29 10.1016/j.cub.2005.12.006 16401415

[B112] Umilta M. A. Kohler E. Gallese V. Fogassi L. Fadiga L. Keysers C. (2001). I know what you are doing: a neurophysiological study. Neuron 31, 155–165 10.1016/S0896-6273(01)00337-3 11498058

[B113] Urgesi C. Maieron M. Avenanti A. Tidoni E. Fabbro F. Aglioti S. M. (2010). Simulating the future of actions in the human corticospinal system. Cereb. Cortex 20, 2511–2521 10.1093/cercor/bhp292 20051359

[B114] Urgesi C. Savonitto M. M. Fabbro F. Aglioti S. M. (2012). Long- and short-term plastic modeling of action prediction abilities in volleyball. Psychol. Res. 76, 542–560 10.1007/s00426-011-0383-y 22045443

[B115] Van den Bussche E. Reynvoet B. (2007). Masked priming effects in semantic categorization are independent of category size. Exp. Psychol. 54, 225–235 10.1027/1618-3169.54.3.225 17725163

[B116] Van den Bussche E. Van den Noortgate W. Reynvoet B. (2009). Mechanisms of masked priming: a meta-analysis. Psychol. Bull. 135, 452–477 10.1037/a0015329 19379025

[B117] Verfaillie K. Daems A. (2002). Representing and anticipating human actions in vision. Vis. Cogn. 9, 217–232 10.1080/13506280143000403

[B118] Wapner S. Cirillo L. (1968). Imitation of a model's hand movements: age changes in transposition of left-right relations. Child Development 39, 887–894 10.2307/1126991 5687333

[B120] Wilson M. Knoblich G. (2005). The case for motor involvement in perceiving conspecifics. Psychol. Bull. 131, 460–473 10.1037/0033-2909.131.3.460 15869341

[B121] Wolpert D. M. Flanagan J. R. (2001). Motor prediction. Curr. Biol. 11, 729–732 10.1016/S0960-9822(01)00432-8 11566114

[B121a] Zwaan R. A. (2004). The immersed experiencer: toward an embodied theory of language comprehension, in The Psychology of Learning and Motivation: Advances in Research and Theory, Vol. 44, ed Ross B. H. (New York, NY: Elsevier Science), 35–62 ISBN: 978-0-12-543344-0

[B122] Zwaan R. A. Taylor L. J. (2006). Seeing, acting, understanding: motor resonance in language comprehension. J. Exp. Psychol. Gen. 135, 1–11 10.1037/0096-3445.135.1.1 16478313

[B123] Zwickel J. Grosjean M. Prinz W. (2007). Seeing while moving: measuring the online influence of action on perception. Q. J. Exp. Psychol. 60, 1063–1071 10.1080/17470210701288722 17654391

